# The defence‐associated transcriptome of hexaploid wheat displays homoeolog expression and induction bias

**DOI:** 10.1111/pbi.12651

**Published:** 2016-11-11

**Authors:** Jonathan J. Powell, Timothy L. Fitzgerald, Jiri Stiller, Paul J. Berkman, Donald M. Gardiner, John M. Manners, Robert J. Henry, Kemal Kazan

**Affiliations:** ^1^ Commonwealth Scientific and Industrial Research Organisation Agriculture St Lucia Queensland Australia; ^2^ Queensland Alliance for Agriculture and Food Innovation University of Queensland St Lucia Queensland Australia; ^3^ Commonwealth Scientific and Industrial Research Organisation Agriculture Black Mountain Australian Capital Territory Australia

**Keywords:** biotic stress, homoeolog expression bias, polyploidy, RNA‐seq, wheat

## Abstract

Bread wheat (*Triticum aestivum* L.) is an allopolyploid species containing three ancestral genomes. Therefore, three homoeologous copies exist for the majority of genes in the wheat genome. Whether different homoeologs are differentially expressed (homoeolog expression bias) in response to biotic and abiotic stresses is poorly understood. In this study, we applied a RNA‐seq approach to analyse homoeolog‐specific global gene expression patterns in wheat during infection by the fungal pathogen *Fusarium pseudograminearum*, which causes crown rot disease in cereals. To ensure specific detection of homoeologs, we first optimized read alignment methods and validated the results experimentally on genes with known patterns of subgenome‐specific expression. Our global analysis identified widespread patterns of differential expression among homoeologs, indicating homoeolog expression bias underpins a large proportion of the wheat transcriptome. In particular, genes differentially expressed in response to *Fusarium* infection were found to be disproportionately contributed from B and D subgenomes. In addition, we found differences in the degree of responsiveness to pathogen infection among homoeologous genes with B and D homoeologs exhibiting stronger responses to pathogen infection than A genome copies. We call this latter phenomenon as ‘homoeolog induction bias’. Understanding how homoeolog expression and induction biases operate may assist the improvement of biotic stress tolerance in wheat and other polyploid crop species.

## Introduction

The vast majority of extant plants species either currently exist in a state of polyploidy (neopolyploidy) or have been affected by polyploidization events during their evolutionary history (paleopolyploidy; Blanc and Wolfe, 2004; Wood *et al*., 2009). Polyploidy occurs via whole‐genome duplication events in the case of autopolyploids or by one or more interspecific hybridization events between different species in the case of allopolyploids (Adams and Wendel, 2005). Polyploid species often display distinct characteristics such as larger seeds (Beaulieu *et al*., 2007) and leaves (Sugiyama, 2005) and more vigorous growth (Ni *et al*., 2009) than their progenitor species. In addition, species with higher states of ploidy often possess better abiotic and biotic stress tolerance than their progenitors (Comai, 2005). Mechanisms of increased biotic and abiotic stress tolerance in polyploid species may involve polyploidy‐contributed heterosis and expression dosage due to increased gene copy number (Chen, 2007).

Polyploidization with its accompanying genomic flux has an enormous impact on global transcriptional regulation relative to patterns of gene expression observed in progenitor species (Chen and Ni, 2006). Accordingly, plants undergo dramatic alterations to global gene expression immediately after a polyploidization event followed by a gradual reversion on an evolutionary timescale to a diploid state (Feldman and Levy, 2009). Without such correction, increased dosage may also be detrimental to plant fitness in newly formed polyploids due to the risk of unbalancing the fine‐tuned regulation of many biological functions in progenitor species (Bekaert *et al*., 2011; Birchler *et al*., 2005). Postpolyploidization events may result in genome asymmetry as homoeologs may be silenced (Sehrish *et al*., 2014) or lost (Schnable *et al*., 2011) or homoeolog expression bias occurs where homoeologs show expression that is different from an assumed equal parental expression ratio (Feldman and Levy, 2009). This process has been shown to occur in a nonrandom fashion, resulting in uneven contribution of particular biological processes and molecular functions from specific subgenomes, a phenomenon termed functional compartmentalization or subfunctionalization (Bekaert *et al*., 2011).

Homoeolog expression biases have been shown to impact on plant growth, development and stress responses in several polyploid species. In allopolyploid cotton, widespread, nonadditive expression patterns and expression partitioning have been identified for homoeologs responding to various abiotic stresses (Dong and Adams, 2011; Liu and Adams, 2007). In newly formed allopolyploid *Arabidopsis*, nonadditive gene expression between homoeologs inherited from *Arabidopsis thaliana* and *A. aerenosa* correlated with transcript instability (Kim and Chen, 2011). Genes with nonadditive expression and associated transcript instability were found to have a strong association with biotic and abiotic stress response gene ontologies (Kim and Chen, 2011). Recent work highlighted a high degree of expression bias and expression partitioning in hexaploid wheat during drought and heat stress (Liu *et al*., 2015).

Comparative transcriptome analyses in polyploids have been utilized to study polyploidy‐associated phenomena in several plant species including the tetraploid cotton (*Gossypium hirsutum*; Flagel *et al*., 2012; Yoo *et al*., 2013) and *Arabidopsis arenosa* (Ng *et al*., 2012; Pignatta *et al*., 2010), hexaploid (bread) wheat (*Triticum aestivum*; Akhunova *et al*., 2010; Leach *et al*., 2014) and dodecaploid common cordgrass (*Spartina anglica*; Chelaifa *et al*., 2010). Akhunova *et al*. (2010) observed a greater contribution to gene expression from the A and B subgenomes compared to the D subgenome in wheat using a homoeolog distinguishing microarray; however, the method utilized in this study was unable to distinguish between A and B homoeologs. Leach *et al*. (2014) utilized RNA‐seq to observe homoeolog expression bias under basal growth conditions in root and shoot tissue for genes occurring on group 1 and group 5 chromosomes of hexaploid wheat. Overall, this study indicated homoeolog expression bias affects a large proportion of the wheat genome but individual subgenomes do not contribute disproportionately to overall homoeolog expression bias, a phenomenon termed ‘balanced homoeolog expression bias’. Approximately 45% of homoeolog triplets displayed expression of all three homoeologs. However, a single homoeolog copy appears to predominantly contribute to the overall transcript abundance (Leach *et al*., 2014). Pfeifer *et al*. (2014) found a high degree of subgenome specialization within the wheat grain transcriptome with particular subgenomes contributing disproportionately to various biological functions including gene expression and translation (A subgenome), cellular macromolecule metabolism (B subgenome) and transport, secretion and communication/signalling (D subgenome).

Previous work has suggested polyploid species tend to exhibit increased tolerance against pathogen attack (Peng *et al*., 2003) and this could at least partly explain early success of neopolyploid species (Oswald and Nuismer, 2007). However, the association between polyploidy and disease resistance can be complex and is not easy to directly test, especially in crop plants where polyploid species have been subjected to artificial selection for resistance while diploid progenitors have not. In other cases, polyploidy may result in increased susceptibility to pathogens if one of the progenitors involved in the polyploid species contains a suppressor or disease susceptibility locus that interfere with the expression of resistance (Kerber, 1991). To date, relatively little work has been performed to assess the effect of biotic stress on genome asymmetry and homoeolog expression bias in polyploid plant species. Previous work in wheat has implicated a primary role for the B subgenome contributing towards biotic stress responses based on distribution of QTL for disease resistance on B subgenome‐specific chromosomes (Feldman *et al*., 2012). The genes involved in the biosynthesis pathway for 2‐benzoxazolinone (BOA), an important phytoalexin, are deployed predominantly from the B subgenome (Nomura *et al*., 2005). Nomura *et al*. (2005) also demonstrated that hexaploid wheat progenitor species (i.e. diploids and tetraploids) are each able to synthesize BOA, suggesting silencing of A and D homoeologs postpolyploidization in hexaploid wheat, resulting in subfunctionalization of BOA synthesis to the B subgenome. Additionally, little work has been performed how genes induced or repressed during a biotic stress response might be biased in responsiveness between homoeologous gene copies. Identifying such ‘homoeolog induction biases’ is also critical to better understanding how each subgenome contributes to biotic stress responses and may shed light on which processes contribute to the success of polyploids against stresses.

In this work, we investigated homoeolog‐specific gene expression patterns of bread wheat infected with *F. pseudograminearum*, a necrotrophic fungal pathogen (Akinsanmi *et al*., 2006), to determine whether different subgenomes respond to pathogen attack differently. *F. pseudograminearum* is the predominant cause of crown rot (Chakraborty *et al*., 2006), a disease with economic significance (Murray and Brennan, 2009) and a highly quantitative basis of resistance (Li *et al*., 2010). Resistance to crown rot in wheat has been previously shown to vary with ploidy level with tetraploid wheat (*T. durum*) displaying greater susceptibility to this pathogen than hexaploid wheat (Liu *et al*., 2012). Previous work has explored wheat responses during *F. pseudograminearum* infection using microarrays (Desmond *et al*., 2008); however, this approach was not able to infer expression patterns in a homoeolog‐specific manner. Our analyses suggest that individual wheat subgenomes contribute disproportionately to the overall response to *F. pseudograminearum* with B and D subgenomes displaying a greater contribution than the A subgenome. Potential implications of this phenomenon on wheat breeding are also discussed.

## Results

### Expanding the known set of homoeolog triplets from the wheat chromosome survey genome sequence using a reciprocal best BLAST analysis

Correctly identifying the full complement of homoeologs in polyploids is essential for homoeolog expression analysis. However, this remains a technical challenge within hexaploid wheat since gene sequence collections are relatively incomplete or contain redundant sequence copies. In order to comprehensively assess potential homoeolog expression bias within the wheat transcriptome, we first aimed to identify as many homoeologous sequences as possible for each wheat gene within the International Wheat Genome Sequencing Consortium (IWGSC) chromosomal survey sequence (CSS) CDS reference (Mayer *et al*., 2014). To do this, we used an approach similar to the one employed by Pfeifer *et al*. (2014) but modified BLAST parameters in an attempt to identify a larger set of homoeologous genes. As explained in Materials and Methods, homoeologous triplets were identified as reciprocal best BLAST (RBB) hits (Moreno‐Hagelsieb and Latimer, 2008) between subgenome‐specific CDS. In order for a homoeolog triplet to be identified, consistent agreement of RBB hits between each subgenome (i.e. A to B, B to D and D to A) is required.

Here, homoeologous triplets were inferred from the global CDS library for 38 889 genes derived from the chromosome arm assemblies (Mayer *et al*., 2014) to form 12 963 triplets corresponding to approximately 39% of CDS in the reference and 28% of total predicted protein coding genes. However, not all homoeolog triplets could be identified mostly due to the absence of a complete reference with gene models. In addition, potential gene deletions and gene duplications producing highly similar sequences could also confound an RBB strategy used for homoeolog identification. Here, we were able to identify 98.7% of all homoeologs previously identified by Pfeifer *et al*. (2014). Furthermore, our analysis identified significantly more homoeolog triplets (12 963) than the analysis in Pfeifer *et al*. (2014) (6576) mainly because we did not apply a minimum BLAST score cut‐off. However, we performed additional analyses to ensure homoeolog triplets were correctly inferred, including confirming that homoeolog chromosomal locations (i.e. 1AL/1BL/1DL) were conserved unless such locations were affected by known translocation events. Overall, we concluded that the number of homoeologs triplets we could identify would be sufficient for global analysis of homoeolog expression patterns in wheat.

### Homoeolog‐specific alignment of RNA‐seq reads validated using differing alignment protocols

To assess homoeolog expression bias in bread wheat during response to biotic stress, an established laboratory infection assay (Yang *et al*., 2010) was performed to infect wheat seedlings with *F. pseudograminearum*. Four biological replicates of *F. pseudograminearum* (*Fp*)‐inoculated and noninoculated (mock) wheat plants were sampled as described in Materials and Methods. We then used RNA‐seq to characterize the transcriptional response in a homoeolog‐specific manner. For each of the approaches described below, reads were aligned to the global coding sequence reference and read counts were then extracted for genes within the inferred homoeolog triplets described in the previous section. For the purpose of testing whether the alignment stringency we used was adequate to differentiate between homoeologs, independent alignments were performed using different methods that employ distinct aligner algorithms. No significant differences in read count estimates were found between heuristic seeding (Bowtie2; Langmead and Salzberg, 2012) versus exhaustive k‐mer (Biokanga; Stephen *et al*., 2012) alignment methods (Appendix S1). No significant differences in read count estimates were observed between random assignment or sloughing of reads which aligned equally well to multiple locations on the reference. To test whether the degree to which homoeologs overlap influences read alignment accuracy, blocks of overlapping coding sequence within homoeolog triplets were retrieved and used as a reference for alignment. From these observations, we concluded that alignment biases should not significantly affect expression estimates for homoeolog triplets where all three sequences are present in the reference used for alignment.

### Homoeolog‐specific alignment stringency validated using for benzoxazolinone biosynthesis pathway

For validating the stringency of the method for estimating transcript abundance in a subgenome‐specific fashion, expression of genes for a well‐characterized phytoalexin biosynthesis pathway with known subgenome‐specific expression was used. As stated above, benzoxazolinone compounds, 2‐benzoxazolinone (BOA) and 6‐methoxy‐benzoxazolinone (MBOA), have been characterized in wheat (Nomura *et al*., 2002). All homoeologous copies of the *TaBx1‐TaBx5* genes involved in BOA biosynthesis in wheat have been identified previously and *Bx* gene expression shown to be predominantly contributed from the B subgenome (Nomura *et al*., 2005). To determine whether we could confirm this finding within our data set, we first used BLAST to identify which sequences within CSS reference correspond to previously characterized wheat *Bx* genes (Appendix S1). Perfect matches were found for all A and D subgenome *Bx* copies and all B subgenome copies except *TaBx3*. The *TaBx3B* sequence (AB042628.1) was added to the reference and read alignment and differential expression analysis were performed again.

This analysis showed that all five *Bx* genes (*TaBx1B*,* TaBx2B*,* TaBx3B*,* TaBx4B* and *TaBx5B*) are highly expressed within mock‐ and pathogen‐infected samples (Appendix S2) with *TaBx4B* and *TaBx5B* significantly repressed by infection (~two‐fold). This observation indicates the RNA‐seq analysis we used was able to distinguish between homoeologs when estimating expression, even for genes such as *Bx*s sharing a high degree of sequence similarity (Nomura *et al*., 2005). From this, we concluded that alignment biases should not significantly affect homoeolog expression estimates provided that all three sequences are present in the reference used for alignment.

### A large degree of homoeolog expression bias occurs during infection

**Figure 1:**
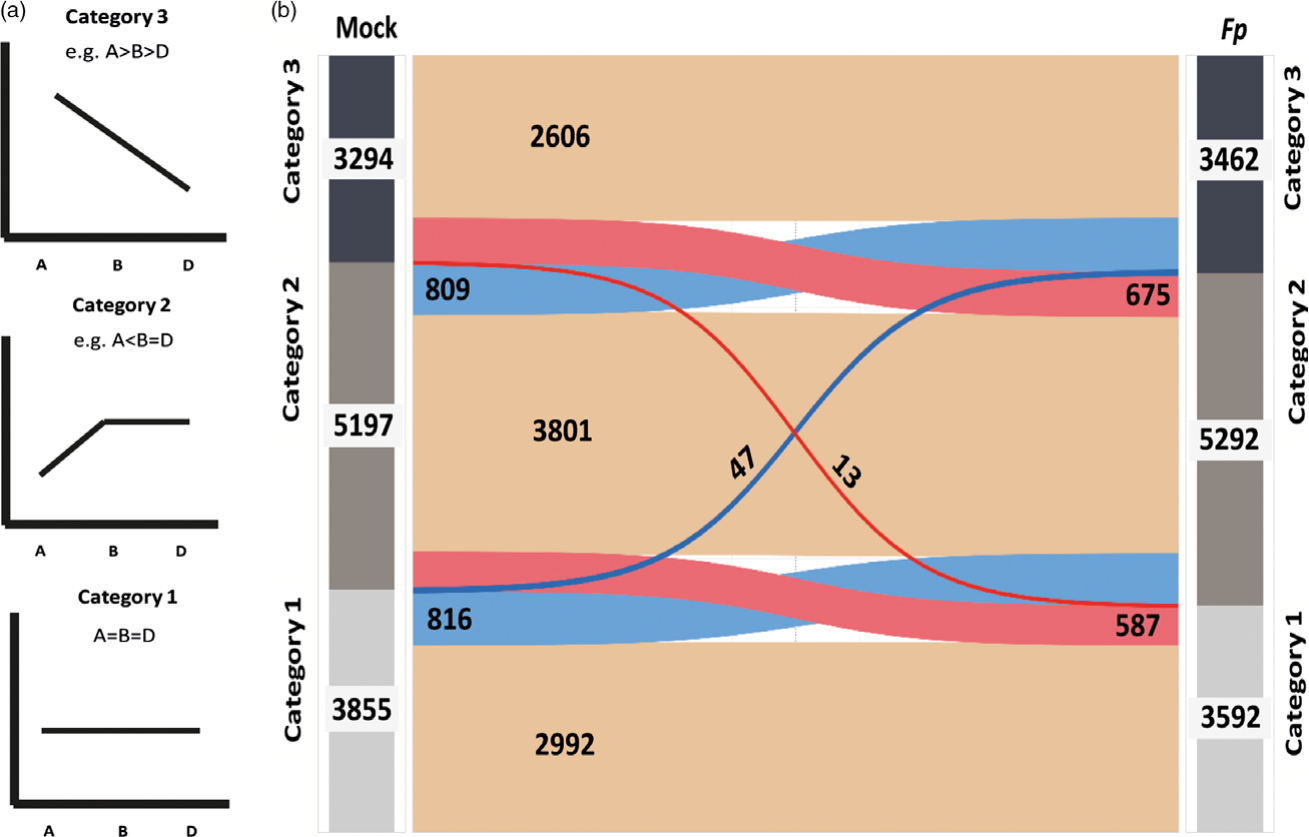
Homoeolog Expression Bias during Biotic Stress. (a) It illustrates three categories of expression pattern identified in this study. Category 1 denotes triplets within which all three homoeologs were expressed to an equivalent level. Category 2 denotes triplets for which one homoeolog was expressed to a significantly different degree compared to both other homoeologs (e.g. A > B = D). Category 3 denotes triplets in which all three homoeologs were significantly differently expressed from each other (e.g. A > B > D). (b) Sankey diagram showing patterns of transition for homoeolog triplets between mock (left side)‐ and *Fp‐*infected (right side) conditions. Beige flows represent homoeolog triplets which retained the same expression pattern under both conditions. Blue flows represent homoeolog triplets which displayed increased expression bias under infected condition relative to mock. Red flows represent homoeolog triplets which displayed reduced expression bias under infected condition relative to mock.

To assess the degree to which homoeolog expression bias occurs within the transcriptome, read counts for A, B and D homoeologs under mock and infected conditions were retrieved from binary alignment map files and used as inputs for DESeq. Genes for which expression of A, B and D homoeologs were not significantly different from each other (A = B = D) were placed into ‘Category 1’. Homoeolog triplets where only one of the homoeolog was differentially expressed (i.e. A > B = D, B > A = D, D > A = B A < B = D, B < A = D or D < A = B) were placed into ‘Category 2’ (Figure 1a). Homoeolog triplets for which expression was significantly different for each homoeolog (i.e. A > B > D, A > D > B, B > A > D, D > A > B, B > D > A and D > B > A) were placed into ‘Category 3’ (Figure 1a). Comparing numbers of homoeolog triplets within Categories 2 and 3 to those in Category 1 provides a measure of the degree of homoeolog expression bias.

For mock‐treated samples, 3855 homoeolog triplets (31%) were assigned to Category 1 while for *Fp*‐treated samples, 3592 homoeolog triplets (29%) were assigned to Category 1 (Figure 1b). Remaining triplets were placed into Category 2 or Category 3; as such, homoeolog expression bias was detected within 69% and 71% of the homoeolog triplets in mock‐ and *Fp*‐treated samples, respectively. The majority of non‐Category 1 homoeolog triplets (5197 mock and 5292 infected) exhibited expression bias towards a single homoeolog (Category 2; Figure 1b). Fewer homoeolog triplets exhibited an unequal expression bias towards two homoeologs (Category 3) with 3294 and 3462 homoeolog triplets identified in mock and infected samples, respectively.

### Patterns of homoeolog expression bias are mostly conserved under infected and uninfected conditions

Understanding how homoeolog biases contribute to responses during infection requires observation of the degree to which homoeolog expression bias patterns are fixed between basal growth and infected conditions and the way in which patterns change during application of a biotic stress. To do this, patterns of expression bias within individual homoeolog triplets were compared under mock and infected conditions (Figure 1). This analysis revealed 2992 homoeologs in total displayed Category 1 expression patterns under both conditions. The majority of triplets showing homoeolog‐specific expression (3801) displayed Category 2 expression patterns under both mock and infected conditions. Significantly fewer homoeologs retained Category 3 expression under both infected and mock conditions (2606; χ^2^ distribution test *P* < 0.01) compared with the number of homoeologs retaining Category 1 and Category 2 expression. For 1672 triplets, the complexity of expression increased under pathogen infection (i.e. 816 triplets from Category 1 to 2, 809 triplets from Category 2 to 3 and 47 triplets Category 1 to 3) while for 1275 triplets, the complexity of expression was decreased under pathogen treatment (587 triplets from Category 2 to 1, 675 triplets from Category 3 to 2 and 13 triplets from Category 3 to 1). Interestingly, where the type of homoeolog expression bias is conserved between mock and pathogen infection (i.e. Category 2 or Category 3 retention), the pattern of expression was also generally conserved (i.e. if A > B > D under mock conditions, then A > B > D under infected conditions as well). A similar trend was observed for homoeolog triplets changing between categories of subgenome expression bias (Category 2 to Category 3 transitions and vice versa) in that the same homoeolog would retain expression bias (i.e. for A > B = D under mock transitioning to A > B > D or A > D > B under infection; Table S1).

### Homoeolog induction bias is a primary driver of subgenomic specificity in the wheat transcriptome

To infer which genes were induced during *Fusarium* infection, differential expression analysis was performed using DESeq. In total, 2755 genes showed significant differential expression in response to *Fusarium* infection at 3 dpi, representing altered expression of approximately 2.8% of the annotated transcriptome (~99K genes). Total differentially expressed genes were comprised of 1867 up‐regulated genes with fold changes ranging from 232 to 1.17. In addition, 888 were down‐regulated under infection (Table 1).

**Table 1:** Table displaying counts of differentially expressed genes globally and for each subgenome specifically in *Triticum aestivum* L. Observations in rows marked by asterisk showed bias from expected proportions as determined by χ^2^ test (*P* < 0.01)

	Global	A genome	B genome	D genome
Up‐regulated genes*	1867	559	639	669
Down‐regulated genes	888	275	306	307
Total*	2755	834	945	976

**Figure 2:**
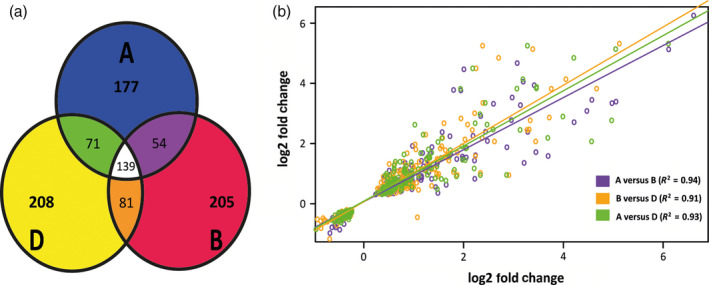
Homoeolog Induction Bias during Biotic Stress. (a) Venn diagram showing counts of differentially expressed genes (*Fusarium* induced) within identified homoeologous triplets. Counts represent triplets where one (no intersection), two (intersection of two circles) or all three (intersection of three circles) genes were differentially expressed. The first two descriptions represent cases of homoeolog induction bias, with the disproportionate indicating homoeolog induction bias strongly underpins the biotic stress‐induced transcriptome. (b) Pairwise correlation expressions for homoeologs differentially expressed from A and B subgenome copies (orange), B and D subgenome copies (purple) and A and D subgenome copies (green).

Within the total set of 1867 up‐regulated genes, 1526 occurred within identified homoeologous triplets. After consolidating homoeologous differentially expressed genes (considering differential expression of multiple homoeolog copies as response of a single gene), the canonical transcriptomic response consisted of 944 genes showing differential expression (62% of all differentially expressed homoeologous triplets). Patterns of differential expression among identified homoeologs revealed 139 triplets where all homoeologs were differentially expressed (417 gene copies in total). We denote instances where one or two homoeologs were differentially expressed between mock and infected conditions as cases of ‘homoeolog induction bias’. Homoeolog induction bias was found to impact a large proportion of the biotic stress‐induced transcriptome. Of 591 genes differentially expressed between mock‐ and pathogen‐inoculated plants in a homoeolog‐specific manner, 54 triplets displayed differential expression of A and B homoeologs only (108 gene copies), 82 from B and D homoeologs only (164 gene copies) and 73 from both A and D homoeologs only (146 gene copies; Figure 2a). A high proportion of biotic stress‐responsive genes were contributed from a single homoeolog copy with 177, 206 and 208 genes and were differentially expressed either from the A, B or D subgenome alone, respectively (Figure 2a). Analysis indicated homoeologs differentially expressed from a single subgenome were disproportionately high (χ^2^ distribution test *P* < 0.01) compared to genes induced from multiple subgenomes. Consequently, single homoeolog expression events also contributed most of the observed functional diversity within the induced transcriptome.

Another potential bias occurring within differentially expressed homoeolog triplets is a bias in the magnitude of induction between homoeologs during infection. To observe whether co‐induced homoeologs differed in magnitude of induction, pairwise comparisons (A vs. B, B vs. D and A vs. D subgenomes) of DE gene fold change values for inferred homoeologs were performed using Spearman's ranking analysis (Figure 2b). Expression fold change values were found to be highly correlated across all three comparisons with an *R* ^2^ value of 0.94 for A subgenome versus B correlation, 0.91 for B versus D correlation and 0.93 for A versus D correlation. When only considering those genes with greater than twofold DE, the degree of correlation was reduced but still significant (*R* ^2^ = 0.72 for A vs. B; 0.66 for B vs. D and 0.61 for A vs. D) with high confidence (*P* < 0.001) across comparisons (Figure 2b). These observations indicate subgenome specificity for response to biotic stress is primarily driven by homoeolog induction bias (i.e. which homoeologs are induced) rather than homoeolog expression bias (i.e. driving the magnitude of induction) when multiple homoeologs in a triplet are differentially expressed.

### Homoeolog expression bias and induction bias impact on biotic stress‐related genes and pathways and establish subgenome specificity

For the purpose of determining whether observed expression and induction biases would involve genes commonly implicated in biotic stress responses, gene descriptions for differentially expressed homoeologs were retrieved using BLAST2GO (Conesa *et al*., 2005). This analysis allowed assignment of functional descriptions for genes that are well known to be involved in pathogen responses such as pathogenesis‐related proteins, leucine‐rich repeat proteins (LRR) and leucine‐rich receptor‐like kinases (LRKs), ABC transporters and pleiotropic drug resistance proteins, germin‐like proteins and glutathione‐S‐transferases (Tables S2 and S3). Contribution of biotic stress‐induced homoeologs was disproportionately contributed (χ^2^ test *P* < 0.05) with pathogenesis‐related proteins and leucine‐rich receptors and kinases contributed more from the D genome compared to A and B and much less frequently from all three subgenomes equally (Table S2).

**Figure 3:**
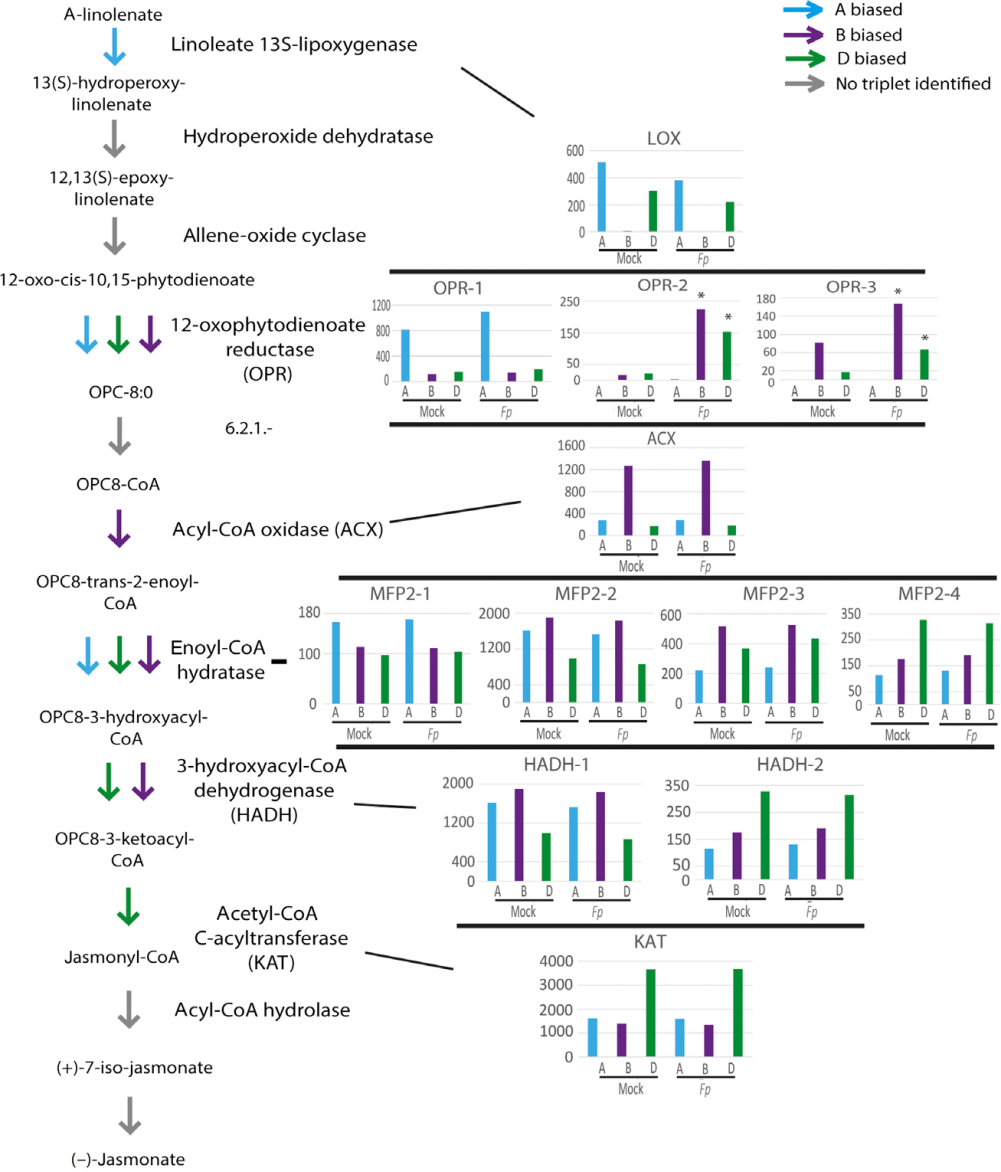
Homoeolog expression bias and induction bias within the jasmonate biosynthesis pathway. Cyan, purple and green arrows represent steps encoded by triplets displaying an expression bias towards the A, B and D subgenome homoeologs, respectively. Grey arrows represent enzymatic steps for which no triplets could be identified. Histograms display read counts for homoeologs under mock‐ and *Fp*‐inoculated conditions and are grouped by corresponding enzyme identity. Homoeologs significantly induced during infection are marked with asterisks.

The plant defence‐associated hormone jasmonic acid is produced from alpha‐linolenate via a series of reactions. In total, 13 homoeolog triplets encoding enzymes in the jasmonate biosynthesis pathway were identified in our analysis. Interestingly, the expression of the gene encoding the lipoxygenase (LOX) enzyme that catalyses the first step in the jasmonate biosynthesis pathway showed a biased expression towards the A subgenome both under mock‐ and pathogen‐inoculated conditions (see inset in Figure 3). The D subgenome also contributes to the LOX expression, but no LOX expression could be detected from the B subgenome under either mock‐ or pathogen‐inoculated conditions (Figure 3 inset). In addition, we identified three paralogous genes (tentatively named as *OPR1*,* OPR2* and *OPR3*), most likely encoding different isoforms of the enzyme 12‐oxophytodienoate reductase (OPR) within each subgenome. Of these three *OPR* genes, *OPR1* expression showed a bias towards the A subgenome while *OPR2* and *OPR3* showed a bias towards B and D subgenomes. In addition, while the biased expression pattern of OPR1 from the A subgenome was not different between pathogen‐ versus mock‐treated samples, B and D homoeolog copies of OPR2 and OPR3 showed a strong induction bias following pathogen inoculation. Similarly, four paralogous genes encoding different isoforms of the enzyme enoyl‐CoA hydratase could be identified within each subgenome. The homoeologs of these paralogous genes showed biased expression towards different subgenomes. Finally, expression patterns of homoeolog genes encoding the enzyme 3‐ketoacyl‐CoA thiolase (KAT) showed a bias towards the D subgenome (Figure 3).

In addition to these defence‐related genes, we identified two defence‐related biosynthetic pathways showing a strong bias towards contribution from B and D subgenomes. Folate (also known as vitamin B) has been shown to be important in SA‐mediated systemic immunity (Wittek *et al*., 2015) and folate starvation has been demonstrated as an important resistance strategy in soya bean against soya bean cyst nematodes (Liu *et al*., 2012). In plants, folate biosynthesis initiates with production of tetrahydrofolate from either guanosine triphosphate or chorismate (Ravanel *et al*., 2001). Tetrahydrofolate then undergoes a series of transformations into five distinct folate derivatives. We were able to identify homoeolog triplets for genes that encode enzymes catalysing the twenty‐four steps in the tetrahydrofolate and folate transformations pathway, while the homoeolog triplets encoding enzymes for the remaining seven steps could not be identified. The genes encoding five of these enzymatic steps showed expression bias towards the B subgenome and eight showed expression bias towards the D subgenome (Appendix S3). Also, for four of these steps, multiple paralogous genes encoding these enzymes within each subgenome were identified. Interestingly, these paralogous genes for a given subgenome homoeolog were found, and these shared the same expression bias pattern. For instance, several paralogous genes (e.g. *MTHFD1.1*,* MTHFD1.2* and *MTHFD1.3*) encoding the enzyme methylenetetrahydrofolate dehydrogenase (EC 1.5.1.5) showed biased expression towards the D subgenome (Appendix S3)

## Discussion

In this study, we utilized an RNA‐seq‐based approach to observe global expression patterns during infection by a pathogen in an unbiased manner and to provide the sensitivity to distinguish between homoeologous copies during read alignment. Past studies examining homoeolog expression patterns in wheat have been restricted by technical limitations inherent to probe‐based methods (Akhunova *et al*., 2010; Leach *et al*., 2014) with more recent approaches adopting RNA‐seq methods to overcome some of these challenges (Pont *et al*., 2011; Pfeifer *et al*., 2014; Nussbaumer *et al*., 2015). RNA‐seq presents a technical improvement over probe‐based transcriptomic analyses since it overcomes the limitation of a finite probe set, an inherent limitation in hybridization‐based detection (Wang *et al*., 2009). In addition, applying RNA‐seq‐based methods to observe the transcriptome in polyploid plants increases the likelihood of detecting homoeolog‐specific polymorphisms, particularly when longer reads are generated (Buggs *et al*., 2012; Higgins *et al*., 2012). Finally, the availability of the IWGSC assembled chromosome sequences in wheat provides a reference for RNA‐seq alignment to allow estimation of gene expression in a chromosome‐ and subgenome‐specific manner (Mayer *et al*., 2014). However, accurate alignment of reads to reference sequence where highly similar sequences exist can still present a technical challenge for any RNA‐seq application. The challenge is compounded in polyploid species by the existence of widespread multiple gene copies with high sequence similarity.

While previous work analysing the transcriptomes of polyploid species has focussed on understanding patterns of expression bias (i.e. the ratio of expression levels for homoeologs) or homoeolog expression dominance (i.e. the level of overall expression compared with the level in progenitor species; Grover *et al*., 2012), there is a paucity of information regarding the degree to which induction profiles across homoeologous copies of biotic stress‐responsive genes differ and whether these induction biases favour particular subgenomes globally. We therefore aimed to examine subgenome‐specific gene expression patterns in bread wheat during fungal infection.

### A high degree of homoeolog expression bias underpins the wheat transcriptome

For differential expression analysis, misalignment biases should affect mock and treated samples equally. However, the accuracy of expression estimates could be affected substantially by misalignment of reads between homoeologs, potentially leading to incorrect inference of homoeolog expression bias. We tested alignment methods with various stringency and ambiguous read handling parameters with results suggesting Burrows–Wheeler transform‐based aligners such as Bowtie2 are able to reliably distinguish between homoeolog copies, consistent with previous findings (Pfeifer *et al*., 2014).

Global observation of expression patterns revealed that homoeolog expression bias underpins a substantial proportion of the wheat transcriptome under both basal growth conditions and increasingly so during infection. Recent work has demonstrated the D subgenome contributes disproportionately to the transcriptional response during infection by *Fusarium graminearum* suggesting the D genome may play a predominant role in responding to this pathogen (Nussbaumer *et al*., 2015). Contribution of homoeolog expression was found to be significantly biased towards B and D subgenomes under both mock and infection conditions consistent with previous findings. This bias may help explain the disproportionate contribution of B and D subgenomes to biotic stress‐responsive genes observed since actively expressed genes are more likely to be induced. In contrast to our findings, previous transcriptome analyses in wheat suggested overall expression bias is balanced across subgenomes (Leach *et al*., 2014; Pfeifer *et al*., 2014). However, in previous studies only a limited number of chromosomes (Group 1 and 5; Leach *et al*., 2014) or a small set of homoeologous loci were examined (Pfeifer *et al*., 2014). Inadequate statistical power of analyses to identify subtle biases may have also been contributed to this discrepancy. Further work is needed to confirm whether the bias we detected towards B and D subgenomes is consistently maintained across environmental conditions and genetic backgrounds. Availability of more powerful statistical analysis methods and the total wheat coding sequence repertoire would allow a greater proportion of homoeolog triplets to be successfully inferred. The retention of homoeolog expression patterns between noninfected and infected conditions suggests for the majority of genes, patterns of expression bias are relatively stable. Therefore, observing homoeolog expression bias under basal conditions may provide an indication of which homoeologs are predominantly expressed under stress‐induced conditions.

Overall, the higher proportion of triplets showing increased expression bias under infection (χ^2^ distribution test *P* < 0.01) suggests biotic stress increases the overall degree of expression bias in the transcriptome; however, a large proportion of triplets displayed the same pattern of expression bias under mock and inoculated conditions. This suggests patterns of subgenome expression bias within homoeolog triplets are generally fixed, but the magnitude of difference tends to increase when biotic stress is applied.

### Subgenome specificity in pathogen response is underpinned by homoeolog induction bias

We describe a new concept for transcriptome analysis in polyploid species which we term ‘homoeolog induction bias’. Homoeolog induction bias differs from homoeolog expression bias as the former considers which homoeolog copies are more responsive during stress conditions rather than differences in magnitude of expression between homoeologs under the same condition. Homoeolog induction bias also differs from expression partitioning (Liu and Adams, 2007) since it does not attempt to explain patterns of induction in the light of shared biological functions or molecular processes, although expression partitioning may often be a strong explanatory factor for biases in homoeolog induction patterns. In this study, the inherent genomic complexity is highlighted in the varied degree to which homoeolog expression bias and homoeolog induction bias were observed within the wheat transcriptome. However, amidst the complexity, evidence for a greater contribution of B and D subgenomes for biotic stress responses emerged.

Here, we demonstrate that a large proportion of the transcriptome diversity in the molecular response to a necrotrophic fungal pathogen is contributed through induction of a single homoeolog. Together with the degree of expression bias favouring a single homoeolog observed, the trend for homoeolog induction bias evinces progress towards functional diploidization in bread wheat (Pfeifer *et al*., 2014). For homoeolog triplets in which two or more homoeologs are actively expressed or induced, it will be interesting to consider why expression of multiple homoeologs is retained in some cases while lost in others. Patterns of retention may be a guided process where increased expression dosage that provides beneficial effects may have been selected for artificially (e.g. through breeding) or naturally. Genes in which expression is only contributed from a single homoeolog are highly attractive targets for knockout, knockdown or mutagenesis approaches to aid functional characterization since this avoids the need to stack multiple altered homoeologs, a time‐consuming and laborious process (Fitzgerald *et al*., 2010, 2015).

### Impact of homoeolog expression bias and induction bias on biotic stress‐related genes

Homoeolog induction for genes associated with biotic stress was generally biased towards B and D subgenomes. The potentially greater importance of the B subgenome in response to pathogens is consistent with the higher proportion of QTL for pathogen resistance occurring on the B subgenome chromosomes than on the other two subgenomes (Feldman *et al*., 2012). In addition, results from our study also suggest a greater contribution of the D subgenome to stress responses than the A subgenome. This suggestion is consistent with the view that the incorporation of the D subgenome in wheat has been a primary driver for the dispersal of bread wheat across temperate agro‐ecological zones (Berkman *et al*., 2013).

The polyploidization history of wheat provides some explanation for the predominant role of B and D subgenomes in biotic stress responses (Marcussen *et al*., 2014). The predominance of the B subgenome over the A subgenome may have resulted through changes to genetic regulation during the period where the progenitor genomes existed in a tetraploid state (Lai *et al*., 2015) driving genome asymmetry in a function‐specific manner. In tetraploid wheat, global transcriptomic analysis revealed the A subgenome to be dominant over the B subgenome in terms of genomic stability (Pont *et al*., 2011). The event in which tetraploid wheat hybridized with the D genome progenitor occurred relatively recently on an evolutionary timescale (Marcussen *et al*., 2014). Thus, codominance of B and D subgenomes in biotic stress response has been maintained over the comparatively short period of hexaploidy. Recent work has demonstrated genome asymmetry patterns vary between natural and synthetic tetraploid wheat genotypes (Wang *et al*., 2016) perhaps suggesting emergence of genome asymmetry following polyploidization occurs in a stochastic rather than directed manner. Further use of synthetic polyploid lines may reveal how genome asymmetry and subfunctionalization between subgenomes occurs.

## Conclusions

Understanding homoeolog expression and induction bias in polyploid crops has critical implications for their genetic improvement, since identification of actively expressed/induced homoeologs will allow targeted inactivation of active homoeologs. Further work studying expression and induction biases across infection time points, tissue types and developmental stages to determine whether expression biases are temporally or spatially determined will provide a more comprehensive understanding of polyploidy‐associated gene expression patterns. Better understanding of how polyploid species utilize their genomic repertoire to endure conditions of stress may enable new strategies to improve agronomic traits in polyploid crops.

## Experimental procedures

### Crown rot infection assay

A soilless infection assay was performed using the commercial wheat (*Triticum aestivum* L.) cultivar ‘Chara’ to observe global transcriptional change during infection by *F. pseudograminearum* isolate CS3427 (CSIRO *Fusarium* collection). *F. pseudograminearum* spores were produced in flask culture using V8 broth (Gardiner *et al*., 2012) by inoculating and incubating on an orbital shaker at room temperature (~22 °C) for 1 week. Spores were harvested by filtering culture through Miracloth (Calbiochem, San Diego, CA) and centrifuging the filtrate in 50‐mL Falcon tubes using a Sigma 4K15 benchtop centrifuge (6000× ***g***) to pellet spores. Spores were resuspended in distilled water to a final concentration of ~1 × 10^6^ spores/mL and stored at −20 °C until required. Seedlings (3 days postgermination) were immersed in *F. pseudograminearum* spores (1 × 10^6^ spores/mL) and incubated for 3 min. Four biological replicates consisting of approximately 12 plants per replicate were included for each treatment to correct for inherent biological variation between plants during infection. To observe transcriptomic change at a relatively early point during infection (prior to visible symptom development), tissue was harvested 3 days postinoculation (dpi) and coleoptile sheath enclosed shoot tissue for each plant was excised and immediately immersed in liquid nitrogen. This was performed to observe response to infection within the crown region with 3 dpi selected as a time point to observe transcriptomic change in response to *F. pseudograminearum* in line with previous work (Desmond *et al*., 2006; Appendix S1).

Validation of successful infection was performed in two ways: firstly, infection replicates were included in the trial and were observed at 14 dpi for development of symptoms (Figure 1). Secondly, cDNA synthesis was performed on aliquots of RNA and relative expression of marker genes for defence responses was assessed using RT‐PCR (Appendix S1). Having observed a strong molecular response at this time point, RNA samples were sent to the Ramaciotti Centre (Sydney, Australia) for library preparation and sequencing.

### Homoeoallele triplet identification

We applied reciprocal best BLAST (RBB; Moreno‐Hagelsieb and Latimer, 2008) between subgenome‐specific coding sequence (CDS) subsets derived from the published wheat chromosome arm assemblies (Mayer *et al*., 2014). RBB hits were identified for each comparison of CDS sets from each of the A vs B, A vs D and B vs D subgenomes. A homoeolog triplet was identified as a set of three genes displaying agreement of genes present in the RBB hits between each pairwise comparison of the subgenome (i.e. A to B, B to D and D to A). Using a custom python script, we compared the homoeoallele triplets identified by our approach with those identified by Pfeifer *et al*. (2014) to determine the level of agreement between the two methods and the number of novel triplets identified by our approach.

### RNA extraction and quality control

RNA extraction was performed using a Qiagen RNeasy extraction kit as per the manufacturer's instructions with the option for on‐column DNase I (Qiagen) digestion. RNA concentration was initially determined using a Nanodrop 2100 spectrophotometer. Integrity of RNA samples was determined using an Agilent Bioanalyser (performed by Australian Genome Sequencing Facility) with all samples having a RIN score >8.5. cDNA synthesis was performed using Invitrogen Superscript III cDNA synthesis kit using oligo‐dT primers to promote transcription of whole mRNA molecules according to manufacturer instructions.

### RNA‐seq library preparation and sequencing

Library preparation and sequencing were performed at the Ramaciotti Centre as described below. RNA was quantified and integrity was assessed a second time prior to sequencing. Sequencing libraries were prepared using standard Illumina library preparation methods. An Illumina HiSeq 2000 platform was used to generate 100‐base pair (bp) paired‐end (PE) reads from isolated mRNA extracted from *F. pseudograminearum* and mock‐infected plants pooled into four biological reps yielding approximately 175 million reads (35gb) in total. Given the estimated size of the bread wheat transcriptome, this represented an average total sequence coverage of ~230x or approximately ~30x per biological sample. Sequence files were deposited to the National Centre for Biotechnology Information (NCBI) Sequence Read Archive under BioProject ID PRJNA297822.

### RNA‐seq analysis

To exclude sequencing errors where possible, sequence quality was analysed using SolexaQA (Cox *et al*., 2010) and paired‐end reads were trimmed to ensure PHRED score >30 prior to alignment (minimum read length 70 bp). Reads were aligned to the *T. aestivum* Chromosomal Survey Sequence (Mayer *et al*., 2014) cDNA collection using Bowtie2 (2.2.3; Langmead and Salzberg, 2012) obtained http://plants.ensembl.org/index.html on 14 May 2014. Paired‐end reads were utilized to help solve ambiguous read alignments. On average, 66% of total reads were successfully aligned to reference across samples. Appendix S1 delineates the process and command lines used within this analysis. Analysis of differential expression was performed using DESeq (Anders, 2010). Homoeolog expression bias was inferred to triplets for which A, B or D homoeologs were differentially expressed under the same condition (adjusted *P* value <0.05 with Bonferroni corrected FDR) and homoeolog induction bias was inferred to triplets in which one or two homoeologs were differentially expressed between mock‐inoculated and *Fusarium*‐inoculated conditions (adjusted *P* value <0.05 with Bonferroni corrected FDR.

A graphical overview of the overall analysis pipeline is provided in Appendix S4.

## Conflict of interest

The authors declare that they have no conflict of interests.

## Supporting information


**Table S1** Expression patterns for homoeolog triplets showing category two expression patterns under mock conditions then transitioning to category three expression patterns during *Fp* infection. Patterns reveal predominantly expressed homoeologs under mock conditions tend to also be predominantly expressed during biotic stress.
**Table S2** Differentially expressed genes observed globally according to biotic stress‐related gene ontologies. Counts show observed number of genes against/expected based on the background number of genes in the annotated reference. Defence genes (PRs and chitinases) and Leucine Rich Repeat proteins were found to have disproportional contribution from subgenomes as determined by χ^2^ test (*P* < 0.01).
**Table S3** Homoeolog triplets with biotic stress‐related gene ontologies, displaying number of ‘A’, ‘B’ and ‘D’ homoeologs which were favoured by expression bias and induction bias.

**Appendix S1** Detailed documentation of experimental approaches and bioinformatic analyses. This file provides additional details on the reference files, software packages and command lines used in this analysis.

**Appendix S2** Testing Alignment Stringency for Differentiating Homoeologous Gene Copies. Panel A: shows expression estimates for all homoeologous copies for Bx1–5 except for the B copy of Bx3 which was found to be absent from the CSS reference. Panel B: shows expression estimates when read alignment was performed using the CSS reference with the known TaBx3B coding sequence added.

**Appendix S3** Homoeolog expression bias within the folate biosynthesis pathway favours B and D subgenomes. Cyan, purple and green arrows represent steps encoded by triplets displaying an expression bias towards the A, B and D subgenome homoeologs, respectively. Grey arrows represent enzymatic steps for which no triplets could be identified and black arrows represent enzymatic steps where triplets showed no bias.

**Appendix S4** Graphical representation of the analysis pipeline to observe homoeolog expression bias during application of a biotic stress. Firstly, an infection assay was performed to produce mock and *F. pseudograminearum* inoculated wheat tissue samples (a) and RNA‐seq was performed to generate reads from transcripts (b). The wheat genome chromosomal survey sequence (coding sequence collection) (c) was utilized within a reciprocal best BLAST approach to identify homoeologs (d) with ~13 000 homoeolog triplets identified and validated (e). RNA‐seq generated reads were aligned to the wheat genome (coding sequences as reference) using Bowtie2 and counted to estimate gene expression globally (f). DESeq was used to identify genes differentially expressed between mock‐ and *Fp*‐treated samples (g). Homoeolog expression bias was assessed in three‐way pairwise comparison using DESeq to identify triplets in which one or more homoeologs were expressed to a significantly different level compared to the others (h). Finally, genes differentially expressed during infection which were also captured within inferred homoeolog triplets were analysed for homoeolog induction bias (i).
