# Supplementary material for: The defence‐associated transcriptome of hexaploid wheat displays homoeolog expression and induction bias
Source: Plant Biotechnol J. 2016 Nov 11;15(4):533–43. doi: 10.1111/pbi.12651 (PMC5362679; doi:10.1111/pbi.12651)
Supplement: Supplementary file 1 — Table S1 Expression patterns for homoeolog triplets showing category two expression patterns under mock conditions then transitioning to category three expression patterns during Fp infection. Patterns reveal predominantly expressed homoeologs under mock conditions tend to also be predominantly expressed during biotic stress. Table S2 Differentially expressed genes observed globally according to biotic stress‐related gene ontologies. Counts show observed number of genes against/expected based on the background number of genes in the annotated reference. Defence genes (PRs and chitinases) and Leucine Rich Repeat proteins were found to have disproportional contribution from subgenomes as determined by χ2 test (P < 0.01). Table S3 Homoeolog triplets with biotic stress‐related gene ontologies, displaying number of ‘A’, ‘B’ and ‘D’ homoeologs which were favoured by expression bias and induction bias. [file PBI-15-533-s004.pptx]

## Slide 1
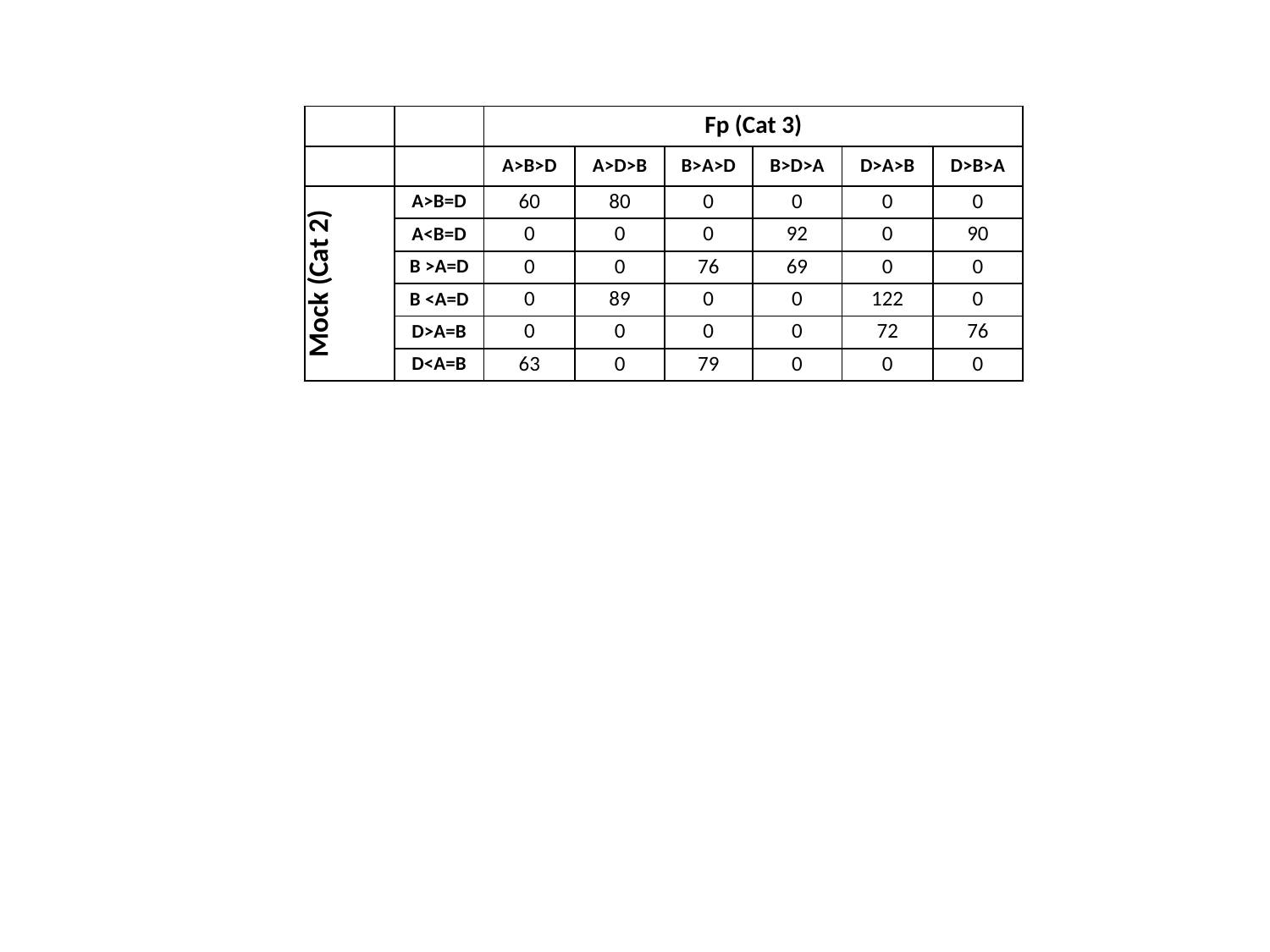

| | | Fp (Cat 3) | | | | | |
| --- | --- | --- | --- | --- | --- | --- | --- |
| | | A>B>D | A>D>B | B>A>D | B>D>A | D>A>B | D>B>A |
| Mock (Cat 2) | A>B=D | 60 | 80 | 0 | 0 | 0 | 0 |
| | A<B=D | 0 | 0 | 0 | 92 | 0 | 90 |
| | B >A=D | 0 | 0 | 76 | 69 | 0 | 0 |
| | B <A=D | 0 | 89 | 0 | 0 | 122 | 0 |
| | D>A=B | 0 | 0 | 0 | 0 | 72 | 76 |
| | D<A=B | 63 | 0 | 79 | 0 | 0 | 0 |

## Slide 2
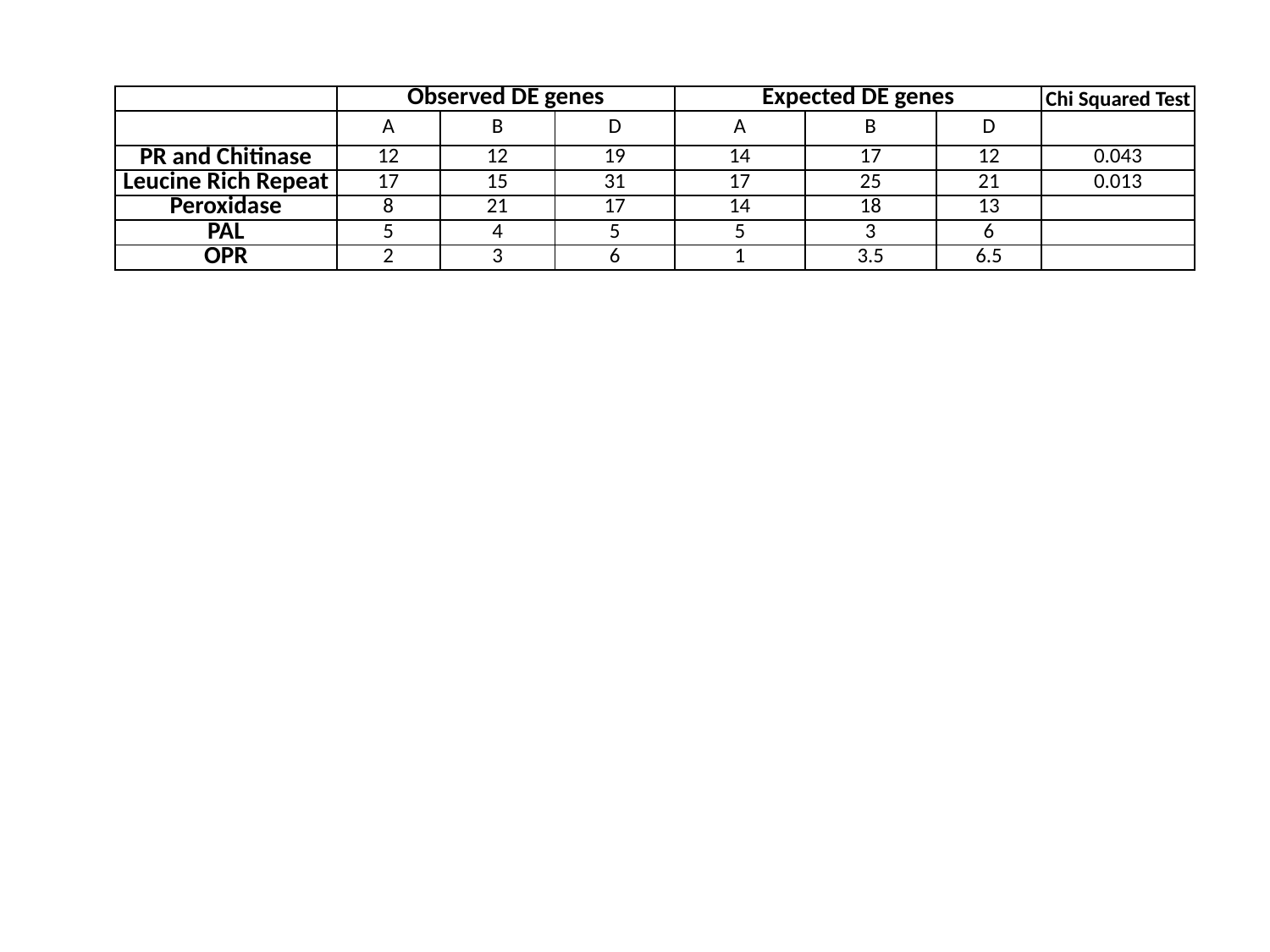

| | Observed DE genes | | | Expected DE genes | | | Chi Squared Test |
| --- | --- | --- | --- | --- | --- | --- | --- |
| | A | B | D | A | B | D | |
| PR and Chitinase | 12 | 12 | 19 | 14 | 17 | 12 | 0.043 |
| Leucine Rich Repeat | 17 | 15 | 31 | 17 | 25 | 21 | 0.013 |
| Peroxidase | 8 | 21 | 17 | 14 | 18 | 13 | |
| PAL | 5 | 4 | 5 | 5 | 3 | 6 | |
| OPR | 2 | 3 | 6 | 1 | 3.5 | 6.5 | |

## Slide 3
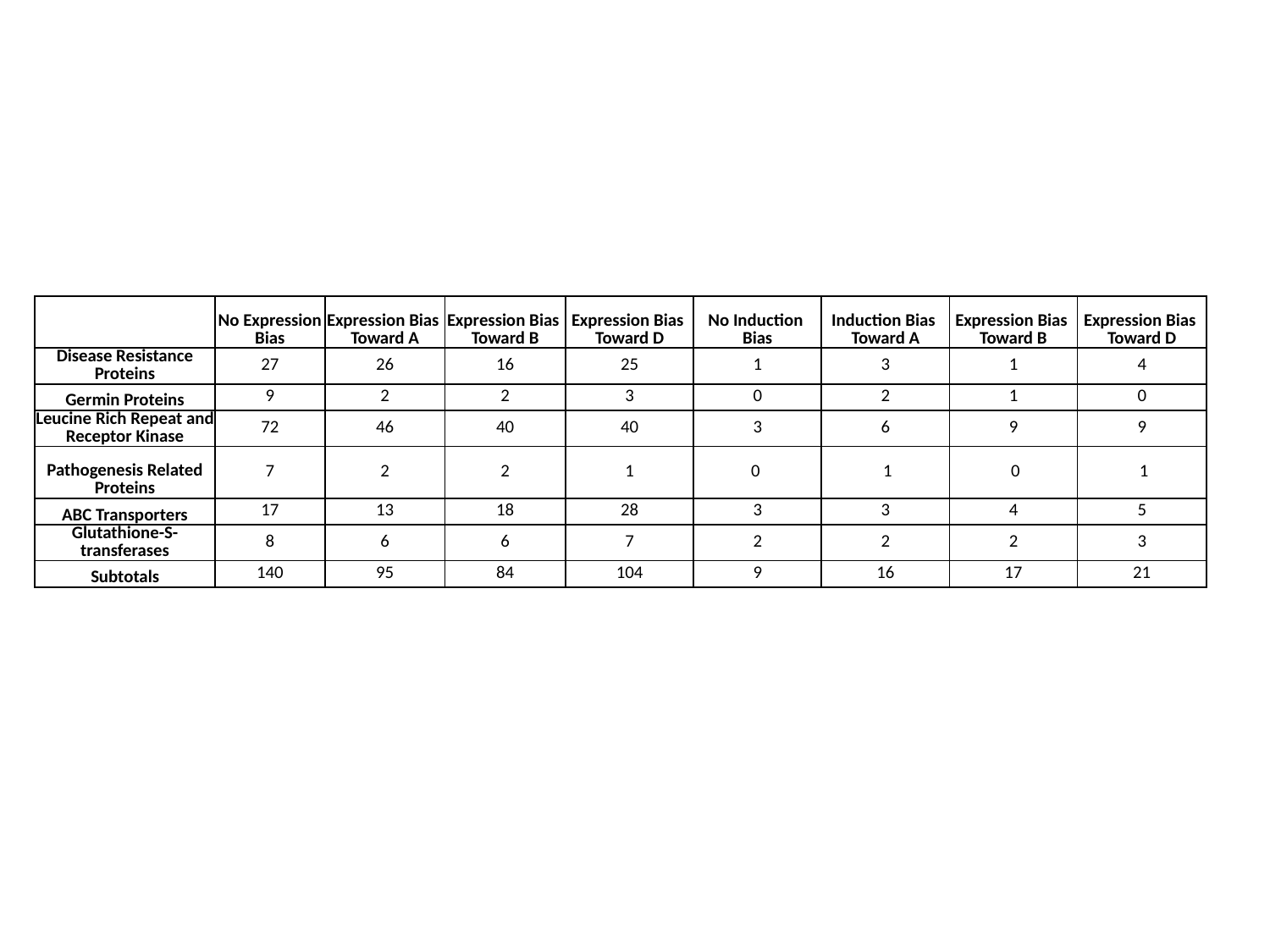

| | No Expression Bias | Expression Bias Toward A | Expression Bias Toward B | Expression Bias Toward D | No Induction Bias | Induction Bias Toward A | Expression Bias Toward B | Expression Bias Toward D |
| --- | --- | --- | --- | --- | --- | --- | --- | --- |
| Disease Resistance Proteins | 27 | 26 | 16 | 25 | 1 | 3 | 1 | 4 |
| Germin Proteins | 9 | 2 | 2 | 3 | 0 | 2 | 1 | 0 |
| Leucine Rich Repeat and Receptor Kinase | 72 | 46 | 40 | 40 | 3 | 6 | 9 | 9 |
| Pathogenesis Related Proteins | 7 | 2 | 2 | 1 | 0 | 1 | 0 | 1 |
| ABC Transporters | 17 | 13 | 18 | 28 | 3 | 3 | 4 | 5 |
| Glutathione-S-transferases | 8 | 6 | 6 | 7 | 2 | 2 | 2 | 3 |
| Subtotals | 140 | 95 | 84 | 104 | 9 | 16 | 17 | 21 |
