# Supplementary material for: The defence‐associated transcriptome of hexaploid wheat displays homoeolog expression and induction bias
Source: Plant Biotechnol J. 2016 Nov 11;15(4):533–43. doi: 10.1111/pbi.12651 (PMC5362679; doi:10.1111/pbi.12651)
Supplement: Supplementary file 2 — Appendix S1 Detailed documentation of experimental approaches and bioinformatic analyses. This file provides additional details on the reference files, software packages and command lines used in this analysis. [file PBI-15-533-s003.docx]

**Supplementary File 1**

**Infection Assay**

To infect wheat seedlings with *Fusarium pseudograminearum*, we utilised an established soil-less infection assay (Li et al. 2010). *Triticum aestivum L.* cv. Chara was selected for this study since it is a cultivar of commercial importance in Australia with large mutant collections previously produced in this background. Wheat seedlings were inoculated with spore suspension produced from *Fusarium pseudograminearum* isolate CS3427. Spores were produced by inoculating V8 juice (1/5 strength) for 4-5 days on an orbital shaker. Spore cultures were then filtered through miracloth (Calbiochem, San Diego, CA) to separate out mycelia and other debris. Flow-through was aliquoted into 50mL Falcon tubes in a swinging bucket centrifuge at 3000rpm (1912rcf) for ten minutes to pellet spores.

Five replicates were included for mock-inoculated and *Fp*-inoculated plants with each replicate comprised of twelve plants. Each replicate included 12 seedlings and was split between three paper towel rolls so that four seedlings were included in each roll. Leaf sheath enclosed tissue was harvested at 3 dpi and snap frozen in liquid nitrogen. A sixth replicate was produced to observe disease progression. Observation of seedlings at 14 dpi indicated a high degree of symptom development indicating the inoculum was highly virulent and infection was successful.


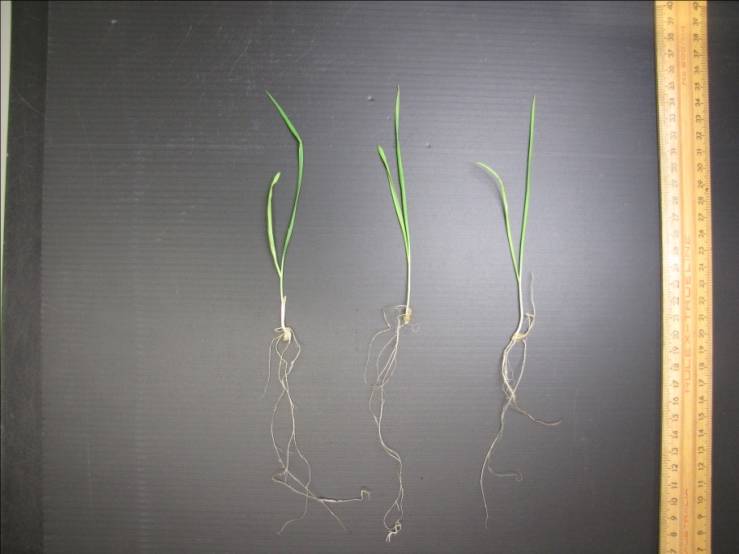

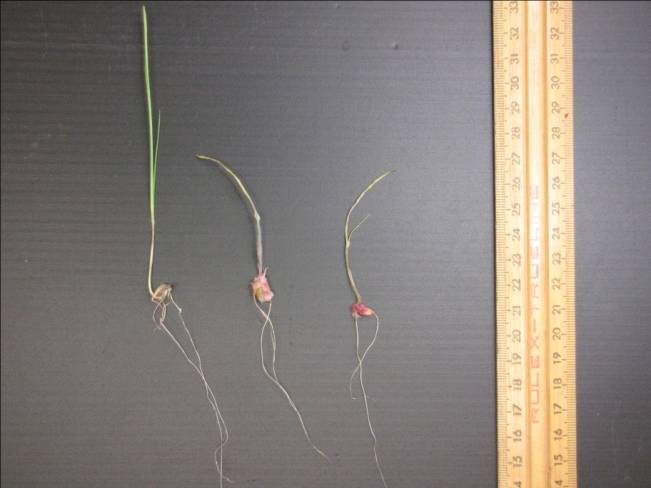


Fp

Mock

Figure 1. Photographs of mock versus *Fusarium pseudograminearum* infected *Triticum aestivum* cv. Chara seedlings at fourteen days post inoculation. Scale bar represents 5 cm.

RNA extractions were performed using Qiagen RNeasy extraction kits with an on-column DNase treatment using the standard plant RNA extraction protocol. cDNA synthesis was performed using Superscript III cDNA synthesis kits. We observed expression of known *Fusarium pseudograminearum* responsive genes assess the degree of infection in *Fusarium* inoculated plants (Desmond et al. 2006). We utilised primer pairs validated by Desmond et al. and reproduce in the table below.

| **Accession** | **Gene name** | **Protein family** | **Primer sequences (5' -3') F** | **Primer sequences (5' - 3') R** |
| --- | --- | --- | --- | --- |
| U76895 | b-tubulin |  | GCCATGTTCAGGAGGAAGG | CTCGGTGAACTCCATCTCGT |
| AJ007348 | PR1.1 | PR1 (basic) | CTGGAGCACGAAGCTGCAG | CGAGTGCTGGAGCTTGCAGT |
| Y18212 | PR2 | PR2 | CTCGACATCGGTAACGACCAG | GCGGCGATGTACTTGATGTTC |
| AF442967 | WAS3a | PR5 | ACAGCTACGCCAAGGACGAC | CGCGTCCTAATCTAAGGGCAG |
| X56011 | TaPERO (Peroxidase) | PR9 | GAGATTCCACAGATGCAAACGAG | GGAGGCCCTTGTTTCTGAATG |
| AJ006098 | Wheatwin 1-2 | PR4 | CGAGGATCGTGGACCAGTG | GTCGACGAACTGGTAGTTGACG |
| U32428 | WCI2 |  | TAGGAACTGGAACTTCACCGAGC | GGTAGTCCTTGATGTGCAGCGAC |
| U32429 | WCI3 |  | AAAGTTGGTCTTGCCACTGACTG | TCGACAAAGCACTTCTGGATTTC |
| AB029934 | Chitinase 1 | PR3 | AGAGATAAGCAAGGCCACGTC | GGTTGCTCACCAGGTCCTTC |
| AJ237942 | TaGLP2a | Germin-like | AACAAAGGTGATGTGTTCGTCTTC | GAGCCGGTCTATTGTATTCTTTTCC |
| CA684431 | PR10 homolog | PR10 | TTAAACCAGCACGAGAAACATCAG | ATCCTCCCTCGATTATTCTCACG |

Table 1. Genes used as markers for host molecular response to infection by real time polymerase chain reaction (RT-PCR). Table gives NCBI accession IDs, gene descriptions, class of defence related proteins, forward and reverse primer sequences. Primer design and validation is outlined in Desmond et al. 2006.

**
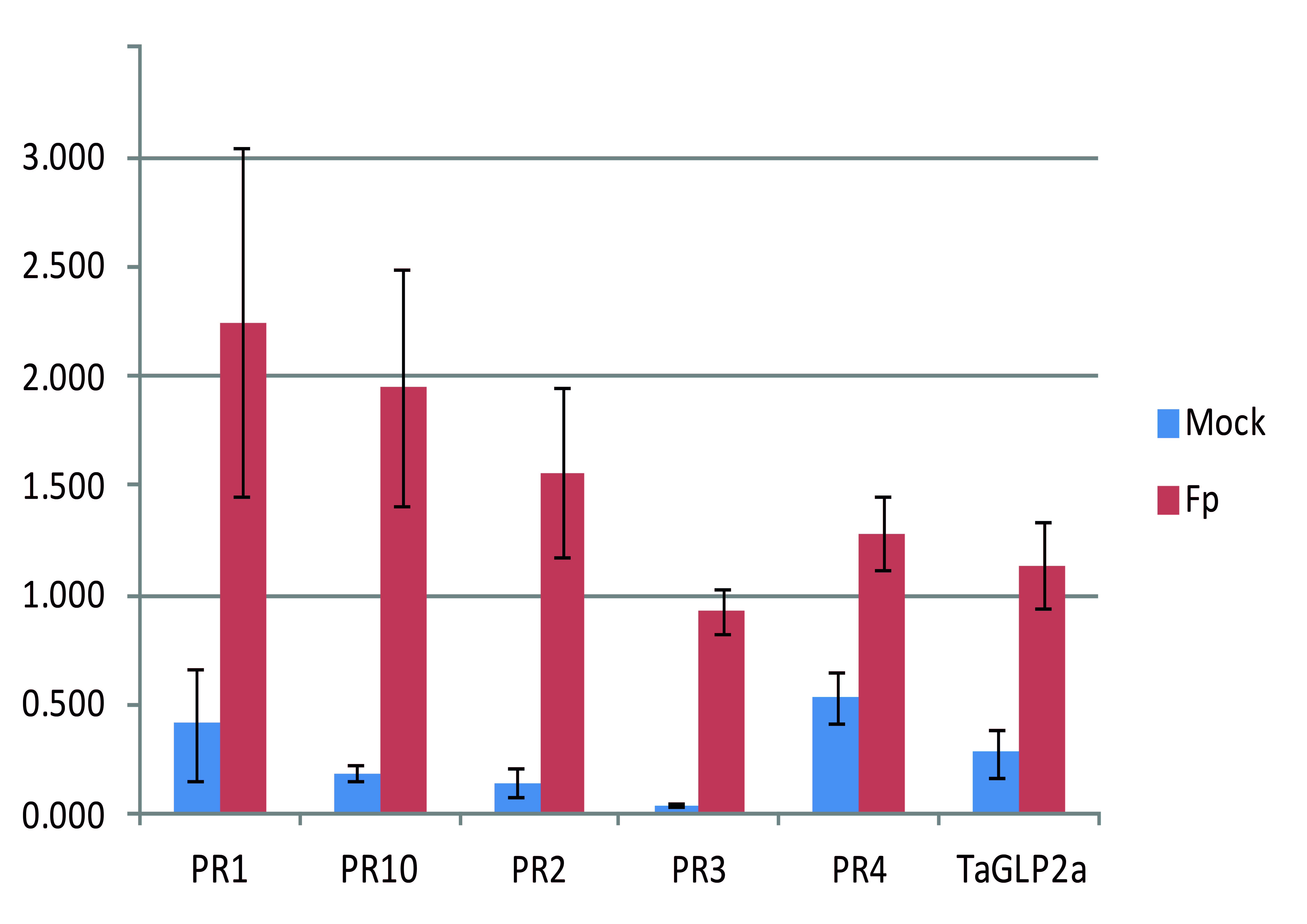
**

**RQ**

Figure 2. Graphs showing expression of a PR1, PR10, PR2, PR3 and a germin-like protein under mock and inoculated conditions within wheat seedlings (normalised to ß-tubulin). These genes serve as markers for response to pathogen infection with the high induction of these genes indicating a high degree of molecular response to infection at the timepoint we selected. Error bars display standard error of the mean. Y-axis represents relative quantitation with *Fp* as the reference biological group (Viia7 software, Applied Biosystems, Waltham, MA) and *Fp*2 as the reference biological sample.

**Identifying Homoeolog Triplets**

Homoeolog triplet identification was performed using a reciprocal best BLAST approach. We took the coding sequence collection and sorted the homoeologs from A, B and D subgenomes into separate files. A custom script was used to retrieve sequences from the CSS CDS multiple FASTA file based on lists of subgenome specific IDs.

retrieve_fasta.pl -f Triticum_aestivum.IWGSP1.22.cdna.all.fa -ids Triticum_aestivum.IWGSP1.22.cdna.all_A_IDs_only.csv

retrieve_fasta.pl -f Triticum_aestivum.IWGSP1.22.cdna.all.fa -ids Triticum_aestivum.IWGSP1.22.cdna.all_B_IDs_only.csv

retrieve_fasta.pl -f Triticum_aestivum.IWGSP1.22.cdna.all.fa -ids Triticum_aestivum.IWGSP1.22.cdna.all_D_IDs_only.csv

Available from GitHub: <https://github.com/jono-powell/wheat_homoeolog_specificity_scripts.git>

From subgenome specific coding sequence FASTA files, we produced BLAST reference databases using the ‘makeblastdb’ package within the BLAST software package.

makeblastdb –in Triticum_aestivum.IWGSP1.22.cdna.all_A.fa -input_type nucl –out \Triticum_aestivum.IWGSP1.22.cdna.all_A.blastdb

makeblastdb –in Triticum_aestivum.IWGSP1.22.cdna.all_B.fa -input_type nucl –out \Triticum_aestivum.IWGSP1.22.cdna.all_B.blastdb

makeblastdb –in Triticum_aestivum.IWGSP1.22.cdna.all_D.fa -input_type nucl –out \Triticum_aestivum.IWGSP1.22.cdna.all_D.blastdb

We then utilised the BLAST algorithm within (software file and version) using the A subgenome sequences as a query to BLAST against the B subgenome generated BLAST database. We performed the reciprocal analysis using B subgenome sequences as a query to BLAST against the A subgenome BLAST database.

blastn -query Triticum_aestivum.IWGSP1.22.cdna.all_A.fa -db Triticum_aestivum.IWGSP1.22.cdna.all_B.blastdb –o Triticum_aestivum.IWGSP1.22.cdna.all_A_vs_ Triticum_aestivum.IWGSP1.22.cdna.all_B.blastdb.blastreport

blastn -query Triticum_aestivum.IWGSP1.22.cdna.all_B.fa -db Triticum_aestivum.IWGSP1.22.cdna.all_A.blastdb –o Triticum_aestivum.IWGSP1.22.cdna.all_B_vs_ Triticum_aestivum.IWGSP1.22.cdna.all_A.blastdb.blastreport

A custom script was used to retrieve all sequences where A and B subgenome sequences formed reciprocal best BLAST hits.

reciprocal_best_blast_hits.pl -a Triticum_aestivum.IWGSP1.22.cdna.all_A_vs_ Triticum_aestivum.IWGSP1.22.cdna.all_B.blastdb.blastreport -b Triticum_aestivum.IWGSP1.22.cdna.all_B_vs_ Triticum_aestivum.IWGSP1.22.cdna.all_A.blastdb.blastreport

Available from GitHub: <https://github.com/jono-powell/wheat_homoeolog_specificity_scripts.git>

The remaining comparisons between B and D and between A and D were made in the same way. Outputs were sorted with Filemaker Pro (relational database software) to identify where unique A, B and D homoeologs were identified consistently as best hits across reciprocal BLAST comparisons.

**Wheat A genome
CDS**

**Wheat B genome
CDS**

**Wheat D genome
CDS**

**RBB**

**RBB**

**RBB**

Figure 3. Graphical representation of the RBB approach used in this study.

**RNAseq analysis**

Sequencing was performed by the Ramaciotti Centre using an Illumina HiSeq Platform to generate 100 base pair paired end reads. Approximately 175 million reads were generated across the eight samples yielding an average of 22 million reads per sample. We utilised a reference based RNAseq analysis approach using Bowtie2 for alignment and DEseq for differential expression analysis (Anders and Huber, 2012). First, we filtered reads using SolexaQA to trim reads to ensure only bases with a minimum PHRED score of 30 were included and a minimum read length of 70 bp was maintained (Cox *et al.*, 2010). We then used Bowtie2 to align trimmed reads to the International Wheat Genome Sequencing Consortium chromosomal survey sequence (CSS) coding sequence collection (CDS).

We produced a bowtie2 reference using the bowtie2-build command.

bowtie2-build Triticum_aestivum.IWGSP1.22.cdna.all.fa Triticum_aestivum.IWGSP1_bowtie2index /Triticum_aestivum.IWGSP1.22.cdna.all

Read alignment was then performed using Bowtie2 to align paired end reads to the global reference.

bowtie2 -x Triticum_aestivum.IWGSP1.22.cdna.all.bowtie2_index/Triticum_aestivum.IWGSP1.22.cdna.all -1 TF_Chara_Fp1_AGTTCC_L007_R1_001.fastq -2 TF_Chara_Fp1_AGTTCC_L007_R2_001.fastq -S TF_Chara_Fp1_AGTTCC_L007_R1_001_vs_Triticum_aestivum.IWGSP1.22.cdna.all_bowtie2.sam --very-sensitive –p 8

*very sensitive uses the stringency settings of -D 20 -R 3 -N 0 -L 20 -i S,1,0.50 where -D gives the number of attempted extensions, -R gives attempted sets of seeds for reads with repetitive seeds, -N = number of mismatches in seed alignment, -L = length of seed substrings and -i interval between seed substrings.

Read alignment presents a potentially confounding technical difficulty in determining accurate gene expression estimates. While Bowtie2 has become a widely used aligner for RNAseq analyses, we were concerned that read alignment handling might lead to significant biases. Bowtie2 utilises a Burrows-Wheeler Transform Alignment algorithm producing small seeding reads (Langmead & Salzberg, 2012). This approach potentially creates a possibility of missing the correct alignment position if the number of seeding sites are exhausted before locating the correct position. BioKANGA is a bioinformatic package produced by the Commonwealth Scientific and Industrial Research Organisation (CSIRO) (Stephen et al. 2012) which utilises K-mer based alignment algorithms rather than Borrows-Wheeler Transform based algorithms. BioKANGA therefore functions as an exhaustive aligner rather than heuristic aligner as is the case for Bowtie2. We performed read alignment with Bowtie2 and BioKANGA aligners independently to assess the degree to aligner algorithm might bias expression estimates. Bowtie2 version 4.7.0 and BioKANGA version 2.62.0 were used.


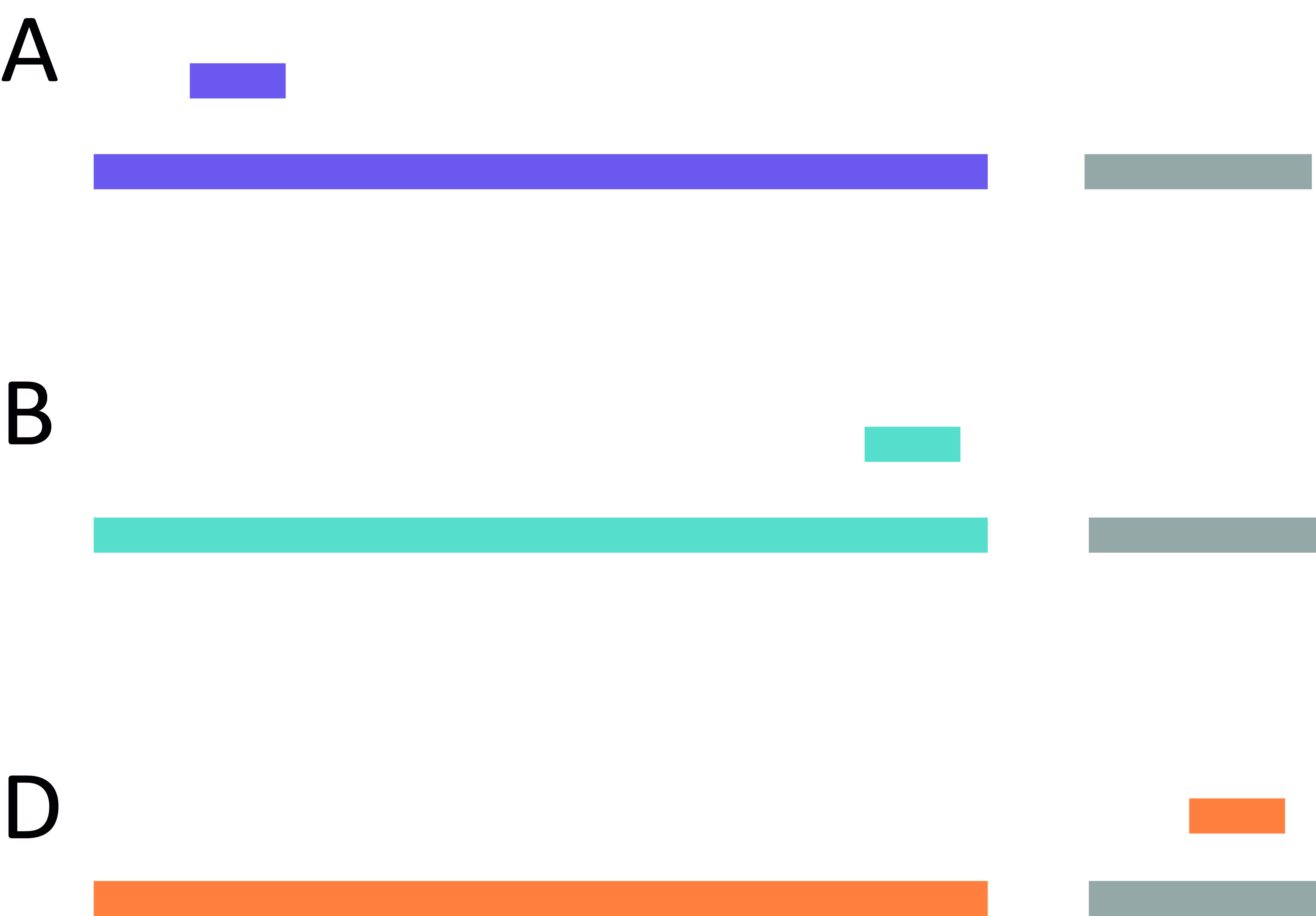


Figure 4. A misalignment scenario in which a read originating from the D homoeolog in this triplet has aligned to highly similar sequence outside the triplet since the heuristic method performed the maximum number of seeds without finding the correct alignment position.

Firstly, we generated a biokanga reference index using the global coding sequence collection.

biokanga index -i Triticum_aestivum.IWGSP1.22.cdna.all.fa -o Triticum_aestivum.IWGSP1.22.cdna.all.biokanga_index -r wheat -T 6

biokanga align –u TF_Chara_Fp1_AGTTCC_L007_R* -I \Triticum_aestivum.IWGSP1.22.cdna.all.biokanga –o TF_Chara_Fp1_AGTTCC_L007_vs_ Triticum_aestivum.IWGSP1.22.cdna.all.sam -T 6 -S CL01_C1FJJACXX_ATCACG_L002_vs_UniGene57K.snp -p 8 -U 2 -s 2

We compared results of differential expression analysis (DEseq) as a measure of similarity in alignment using different alignment algorithms. We found similar numbers and a high degree of overlap of significantly differentially expressed genes using bowtie2 and biokanga. The similarity in results indicates use of heuristic Burrows Wheeler Transform methods does not produce a significant degree of misalignment in a complex polyploid reference.

Another potential technical difficulty lies in the way aligners deal with reads which align equally well to multiple locations on the reference sequence. Bowtie2, by default, will randomise reads which align equally well to multiple locations between those locations. This could potentially lead to inaccuracies in gene expression estimates, particularly in cases where highly similar sequences exist such as homoeologous and paralogous gene copies. In order to assess the degree to which random assignment of reads biases differential gene expression, we utilised the different read handling options in BioKANGA align to compare results when applying random assignment of reads or sloughing reads which align equally well to multiple reference locations. We also compared scenarios under high stringency versus relaxed stringency parameters whether a maximum of 2 or 5 substitutions were permitted for alignment.


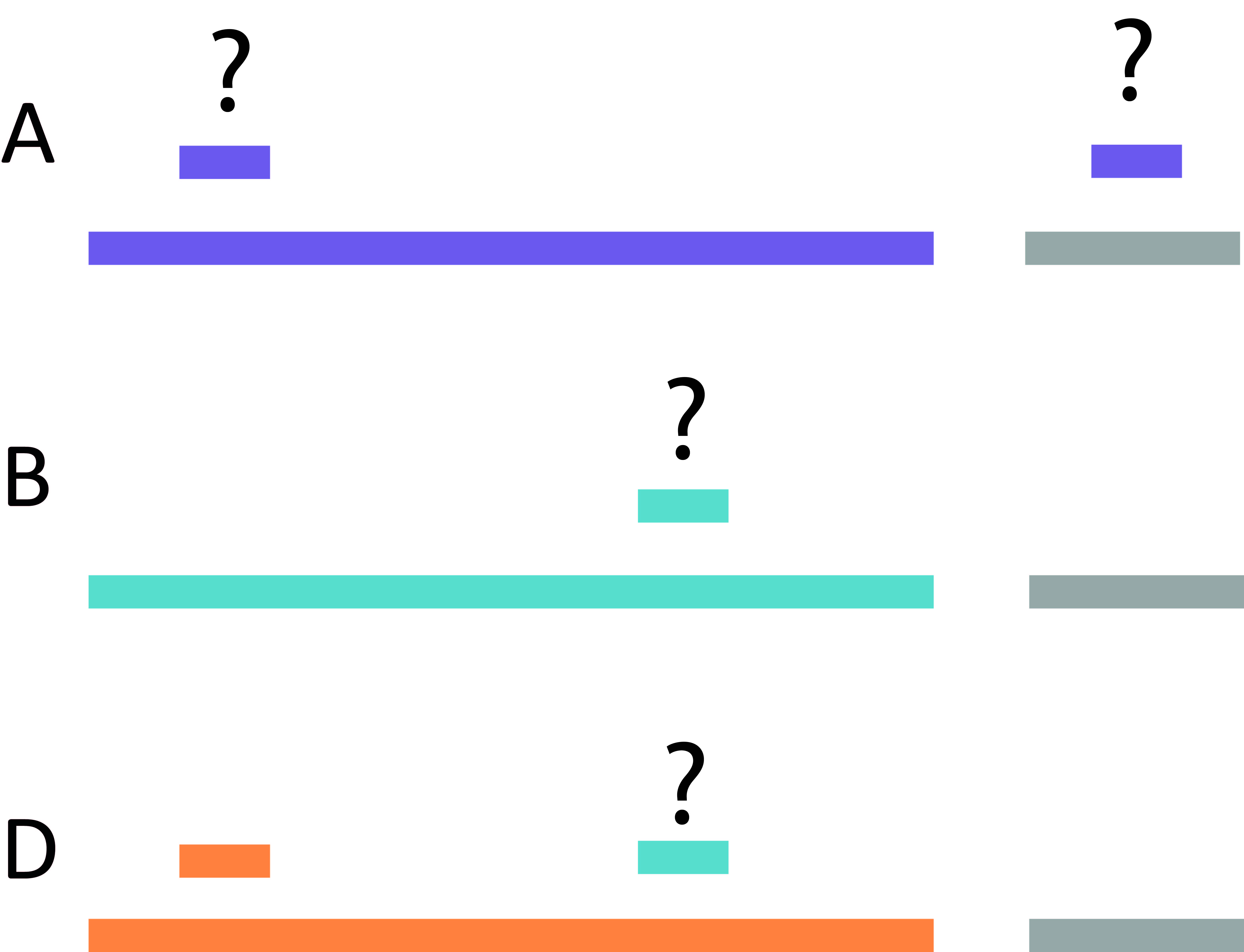


Figure 5. A misalignment scenario in which the aligner identifies multiple, equally good alignments for reads and randomly assigns reads between alignment options.

biokanga align -u TF_Chara_Fp1_AGTTCC_L007_R* -I \Triticum_aestivum.IWGSP1.22.cdna.all.biokanga -o \biokanga\cds\s_2\TF_Chara_Fp1_AGTTCC_L007_R1_001_vs_Triticum_aestivum.IWGSP1.22.cdna.all_bowtie2.sam –U 2 -r 0 -s 2

biokanga align -u TF_Chara_Fp1_AGTTCC_L007_R* -I \Triticum_aestivum.IWGSP1.22.cdna.all.biokanga -o \biokanga\cds\s_2\TF_Chara_Fp1_AGTTCC_L007_R1_001_vs_Triticum_aestivum.IWGSP1.22.cdna.all_bowtie2.sam –U 2 -r 2 -s 2

biokanga align -u TF_Chara_Fp1_AGTTCC_L007_R* -I \Triticum_aestivum.IWGSP1.22.cdna.all.biokanga -o \biokanga\cds\s_2\TF_Chara_Fp1_AGTTCC_L007_R1_001_vs_Triticum_aestivum.IWGSP1.22.cdna.all_bowtie2.sam –U 2 -r 0 -s 5

biokanga align -u TF_Chara_Fp1_AGTTCC_L007_R* -I \Triticum_aestivum.IWGSP1.22.cdna.all.biokanga -o \biokanga\cds\s_2\TF_Chara_Fp1_AGTTCC_L007_R1_001_vs_Triticum_aestivum.IWGSP1.22.cdna.all_bowtie2.sam –U 2 -r 2 -s 5

We compared results of differential expression analysis (DEseq) as a measure of similarity in alignment between under different stringency conditions and read handling methods. The number of differentially expressed genes and overlap in identified differentially expressed genes between comparisons were highly similar across stringency and multiply aligned read handling options.

Another potential technical challenge exists due to the incompleteness of the reference sequences within the coding sequence collection. In cases where differences in sequence overlap occurs between homoeologs, biases could be produced where reads generated for a homoeolog region absent within the reference may misalign to homoeologous regions present within the reference.


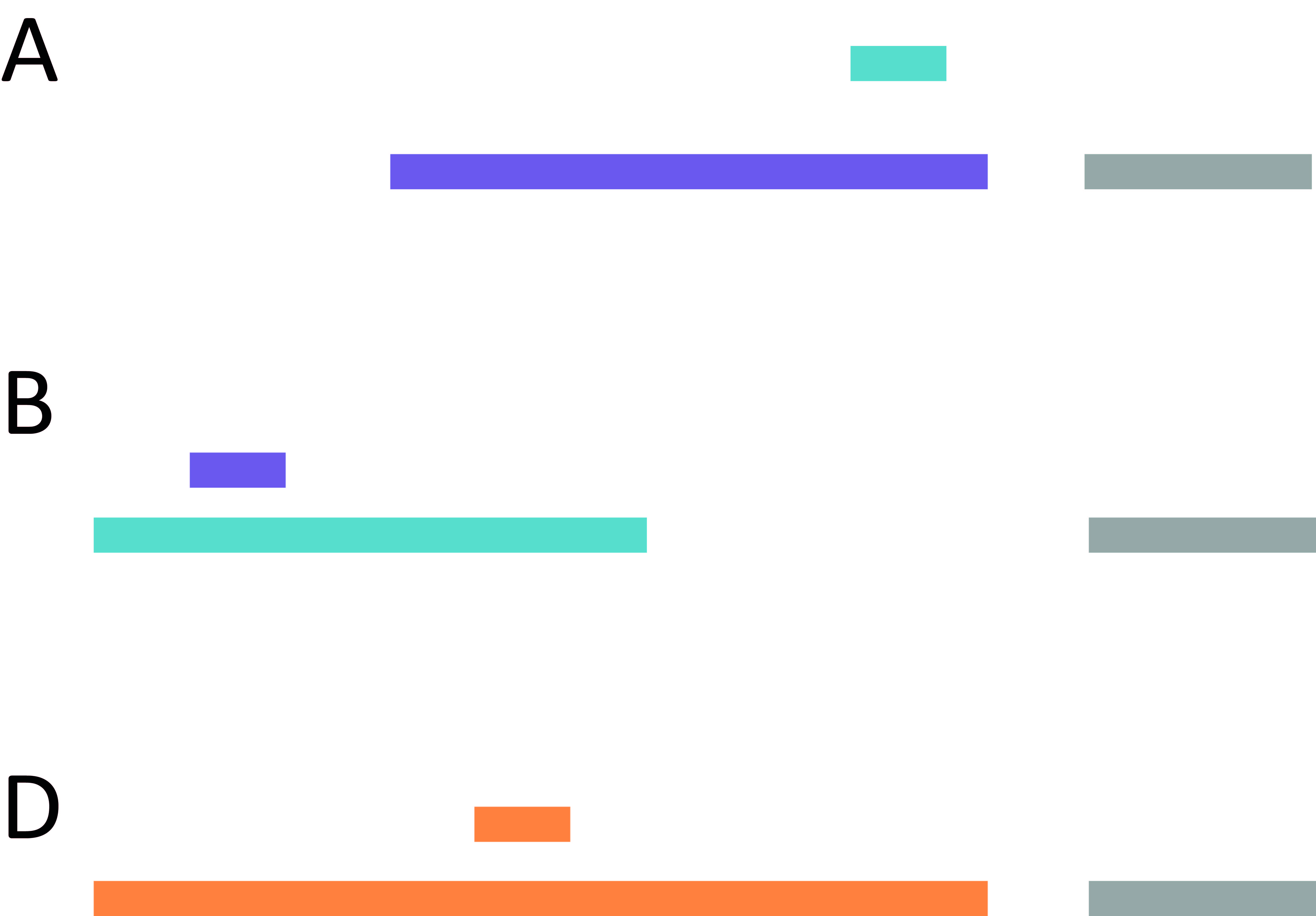


Figure 6. A misalignment scenario in which reads align to the incorrect homoeolog copies because the correct alignment region was absent from the reference.

GBlocks utilises a ClustalW algorithm to align and select regions with high sequence similarity. We used identified sequences from identified homoeolog triplets as inputs to identify conserved sequence between the identified set of homoeolog triplets. From the gblocks analysis, 12776 homoeolog triplets had a conserved region with a minimum of 200bp across all three triplets.

From the Gblocks alignments, we produced a new reference file from sequences conserved between all three homoeologs for each homoeolog triplet. A custom script was used to retrieve conserved regions from each gblocks alignment.

pipeline_allhits.pl -dir ./gblock -cons_min 200 -del

Available from GitHub: <https://github.com/jono-powell/wheat_homoeolog_specificity_scripts.git>

These conserved regions were then concatenated into a single reference file. We then performed a series of alignments using BioKANGA align with the new file as a reference.

biokanga index -i Triticum_aestivum.IWGSP1. homoeologs.conserved.cds.fa -o Triticum_aestivum.IWGSP1.homoeologs.conserved.cds.biokanga -r wheat -T 6

biokanga align -u TF_Chara_Fp1_AGTTCC_L007_R* -I Triticum_aestivum.IWGSP1.homoeologs.conserved.cds.biokanga -o \biokanga\cds\s_2\TF_Chara_Fp1_AGTTCC_L007_R1_001_vs_Triticum_aestivum.IWGSP1.homoeologs.conserved.cds.biokanga.sam –U 2 -r 0 -s 2

biokanga align -u TF_Chara_Fp1_AGTTCC_L007_R* -I Triticum_aestivum.IWGSP1.homoeologs.conserved.cds.biokanga -o \biokanga\cds\s_2\TF_Chara_Fp1_AGTTCC_L007_R1_001_vs_Triticum_aestivum.IWGSP1.homoeologs.conserved.cds.biokanga.sam –U 2 -r 0 -s 5

biokanga align -u TF_Chara_Fp1_AGTTCC_L007_R* -I Triticum_aestivum.IWGSP1.homoeologs.conserved.cds.biokanga -o \biokanga\cds\s_2\TF_Chara_Fp1_AGTTCC_L007_R1_001_vs_Triticum_aestivum.IWGSP1.homoeologs.conserved.cds.biokanga.sam –U 2 -r 2 -s 2

biokanga align -u TF_Chara_Fp1_AGTTCC_L007_R* -I Triticum_aestivum.IWGSP1.homoeologs.conserved.cds.biokanga -o \biokanga\cds\s_2\TF_Chara_Fp1_AGTTCC_L007_R1_001_vs_Triticum_aestivum.IWGSP1.homoeologs.conserved.cds.biokanga.sam –U 2 -r 0 -s 5

We compared results of differential expression analysis (DEseq) as a measure of similarity in alignment between under different stringency conditions. Again, the number of differentially expressed genes and overlap in identified differentially expressed genes between comparisons were highly similar across stringency and multiply aligned read handling options.

***Estimating Transcript Abundance***

Since expression was estimated as read counts, we utilised DEseq as described in (Anders 2010). Firstly, we used the maploci package within the BioKANGA software to produce read counts from sequence alignment maps.

BioKANGA maploci command lines:

biokanga maploci -i ../Chara_Mock${X}_vs_Triticum_aestivum.IWGSP1.22.cdna.all_bowtie2.sam -I ../../../../wheat_sequence/CSS/Triticum_aestivum.IWGSP1.22.cdna.all.bed -o Chara_Mock${X}_vs_Triticum_aestivum.IWGSP1.22.cdna.all_bowtie2.results -O Chara_Mock${X}_vs_Triticum_aestivum.IWGSP1.22.cdna.all_bowtie2.features_results

for X in {1..4}; do biokanga maploci -i ../Chara_Fp${X}_vs_Triticum_aestivum.IWGSP1.22.cdna.all_bowtie2.sam -I ../../../../wheat_sequence/CSS/Triticum_aestivum.IWGSP1.22.cdna.all.bed -o Chara_Fp${X}_vs_Triticum_aestivum.IWGSP1.22.cdna.all_bowtie2.results -O Chara_Fp${X}_vs_Triticum_aestivum.IWGSP1.22.cdna.all_bowtie2.features_results

We then generated DEseq input files using the genDEseq module within BioKANGA.

genDESeq -t Mock -T Fp -iChara_Mock*_vs_Triticum_aestivum.IWGSP1.22.cdna.all_bowtie2.features_results -IChara_Fp*_vs_Triticum_aestivum.IWGSP1.22.cdna.all_bowtie2.features_results -o Chara_Mock_vs_Fp_3DPI_Triticum_aestivum.IWGSP1.22.cdna.all_bowtie2_genDESeq.genes

Finally, we performed differential expression analysis using DEseq.

~/apps/trinity/trinityrnaseq_r2013-02-25/Analysis/DifferentialExpression/run_DE_analysis.pl --matrix Chara_Mock_vs_Fp_3DPI_Triticum_aestivum.IWGSP1.22.cdna.all_bowtie2_genDESeq.genes --method DESeq --samples_file samples_file

**
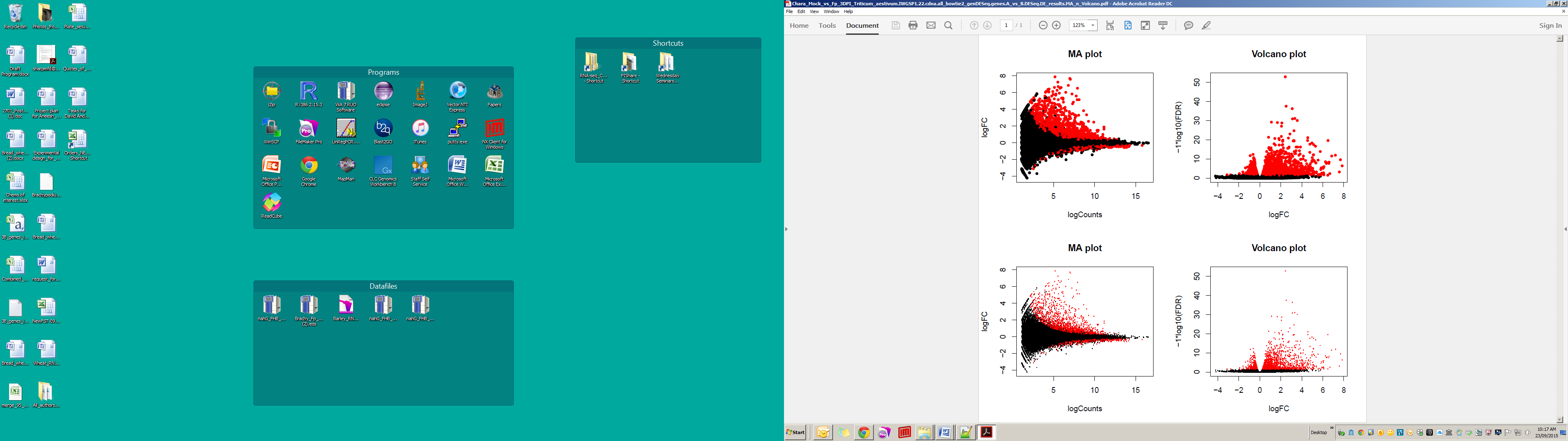
**

Figure 5. Minus average plot showing log fold-change values against log read counts for each gene. Volcano plot showing confidence for differential expression
 (-1*log10(FDR)) against degree of differential expression (log fold change).

**Benzoxazalinone Biosynthesis Gene Identification**

To the best of the authors’ knowledge, relatively few wheat genes with consistent, subgenome specific expression patterns have been identified and other genes with known subgenome specific expression such as bread quality genes (e.g. pinA) are not expressed in shoot/leaf tissue. As such, the benzoxazolinone pathway presents as an ideal test case since it is strongly expressed in seedling shoot tissue and has known, strong subgenome specific expression.

To identify genes encoding enzymes in the benzoxazolinone pathway within the CSS CDS reference, we retrieved known coding sequences for these genes from the NCBI database.

Table 2 *Triticum aestivum* Bx Gene IDs, accessions and corresponding sequence IDs.

| **Gene** | **Accession ID** | **Gene Description** | **CSS Sequence ID** | **Seq Length** | **E value** |
| --- | --- | --- | --- | --- | --- |
| *TaBx1A* | AB094060 | *Triticum aestivum* TaBx1A mRNA | Traes_4AS_9DCDB9778.1 |  | 0 |
| *TaBx1B* | AB124849 | *Triticum aestivum* TaBx1B mRNA | Traes_4BL_4AACA4A08.2 |  | 0 |
| *TaBx1D* | AB124850 | *Triticum aestivum* TaBx1A mRNA | Traes_4DL_B7E95BC28.1 |  | 0 |
|  |  |  |  |  |  |
| *TaBx2A* | AB042630.1 | *Triticum aestivum* CYP71C9v1 mRNA | Traes_4AS_B059A2469.1 | 1930 | 0 |
| *TaBx2B* | AB042631.1 | *Triticum aestivum* CYP71C9v2 mRNA | Traes_4BL_42305EC28.1 | 3291 | 0 |
| *TaBx2D* | AB124851.1 | *Triticum* *aestivum* CYP71C9v3 mRNA | Traes_4DL_B2EA1B486.1 | 3278 | 0 |
|  |  |  |  |  |  |
| *TaBx3A* | AB042628.1 | *Triticum* *aestivum* CYP71C7v2 mRNA | Traes_5AS_B85789CFB.1 | 2272 | 0 |
| *TaBx3B* | - | - | - | - | - |
| *TaBx3D* | AB124852.1 | *Triticum* *aestivum* CYP71C7v1 mRNA | Traes_5DS_B9BFD5BEC.1 | 3062 | 0 |
|  |  |  |  |  |  |
| *TaBx4A* | AB124854.1 | *Triticum* *aestivum* CYP71C6v2 mRNA | Traes_5AS_D06CE8CE5.1 | 3306 | 0 |
| *TaBx4B* | AB124855.1 | *Triticum* *aestivum* CYP71C6v3 mRNA | Traes_5BS_197E3DE21.1 | 3373 | 0 |
| *TaBx4D* | AB042627.1 | *Triticum* *aestivum* CYP71C6v1 mRNA | Traes_5DS_A6848AB19.1 | 3336 | 0 |
|  |  |  |  |  |  |
| *TaBx5A* | AB124856.1 | *Triticum* *aestivum* CYP71C8v1 mRNA | Traes_5BS_51316B1B6.1 | 3434 | 0 |
| *TaBx5B* | AB042629.1 | *Triticum* *aestivum* CYP71C8v2 mRNA | Traes_5DS_6A3EB1F87.1 | 3016 | 0 |
| *TaBx5D* | AB124857.1 | *Triticum* *aestivum* CYP71C8v3 mRNA | Traes_5DS_6A3EB1F87.1 | 3334 | 0 |

We produced a BLAST database from the CDS coding sequence collection in order to provide a reference to BLAST the known Bx gene sequences against.

makeblastdb –in Triticum_aestivum.IWGSP1.22.cdna.all.fa -input_type nucl –out Triticum_aestivum.IWGSP1.22.cdna.all.blastdb

We then used each sequence as a BLAST query against the CSS CDS BLAST database. This approach returned a best BLAST match with perfect identity for three homoeologous copies of each Bx gene (Bx1-5) with the exception of TaBx3B

blastn -query Triticum_aestivum_Bx_coding_sequences.fa -db Triticum_aestivum.IWGSP1.22.cdna.all.blastdb –o Triticum_aestivum_Bx_coding_sequences_vs_ Triticum_aestivum.IWGSP1.22.cdna.all.blastdb.blastreport

These sequences were then analysed for subgenome specific expression, finding a strong bias towards contribution from B subgenome homoeologs.

**Homoeolog Expression Bias and Homoeolog Induction Bias Testing**

Homoeolog expression bias and homoeolog induction bias were assessed among homoeolog triplets. Read counts were retrieved for homoeologs which were able to be assigned into triplets using relationship database software (Filemaker Pro). To identify triplets displaying homoeolog expression bias, pairwise testing (A : B, B : D and A : D) was applied using DESeq employing multiple comparisons correction (Bonferroni) with a significance threshold of adjusted P value < 0.05.

Patterns of homoeolog expression were defined in three categories: category one where each homoeolog is expressed to a statistically equivalent level [no expression bias], category two where one homoeolog was significantly differently expressed to the other two (which were equivalent) [simple expression bias] and category three where each homoeolog was significantly differently expressed to the others [complex expression bias]. Homoeolog triplets were assigned to category one if either none or one of the tests showed a significant difference, category two if two tests showed a significant difference and category three if all three tests returned a significant result. Examples of category two expression patterns are displayed in the figure below. Six potential patterns of bias are possible expressed as A>B=D, A<B=D, B>A=D, B<A=D, D>A=B and D<A=B.


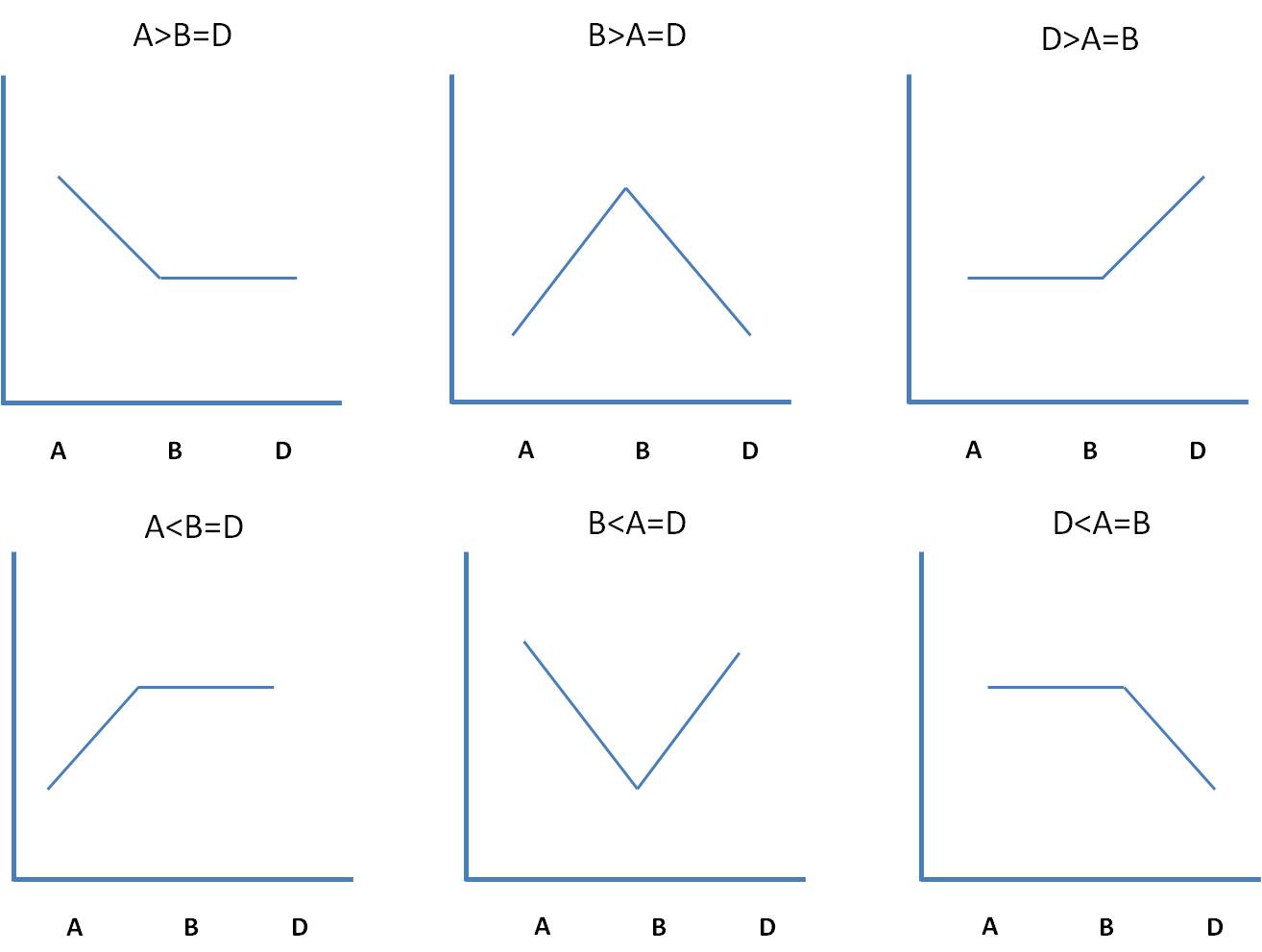


Fig 6. Examples of category two expression patterns. Six potential patterns of bias are possible expressed as A>B=D, A<B=D, B>A=D, B<A=D, D>A=B and D<A=B.

Examples of category three expression patterns are displayed in the figure below. Six potential patterns of bias are possible expressed as A>B>D, A>D>B, B>A>D, B>D>A, D>A>B and D>B>A.


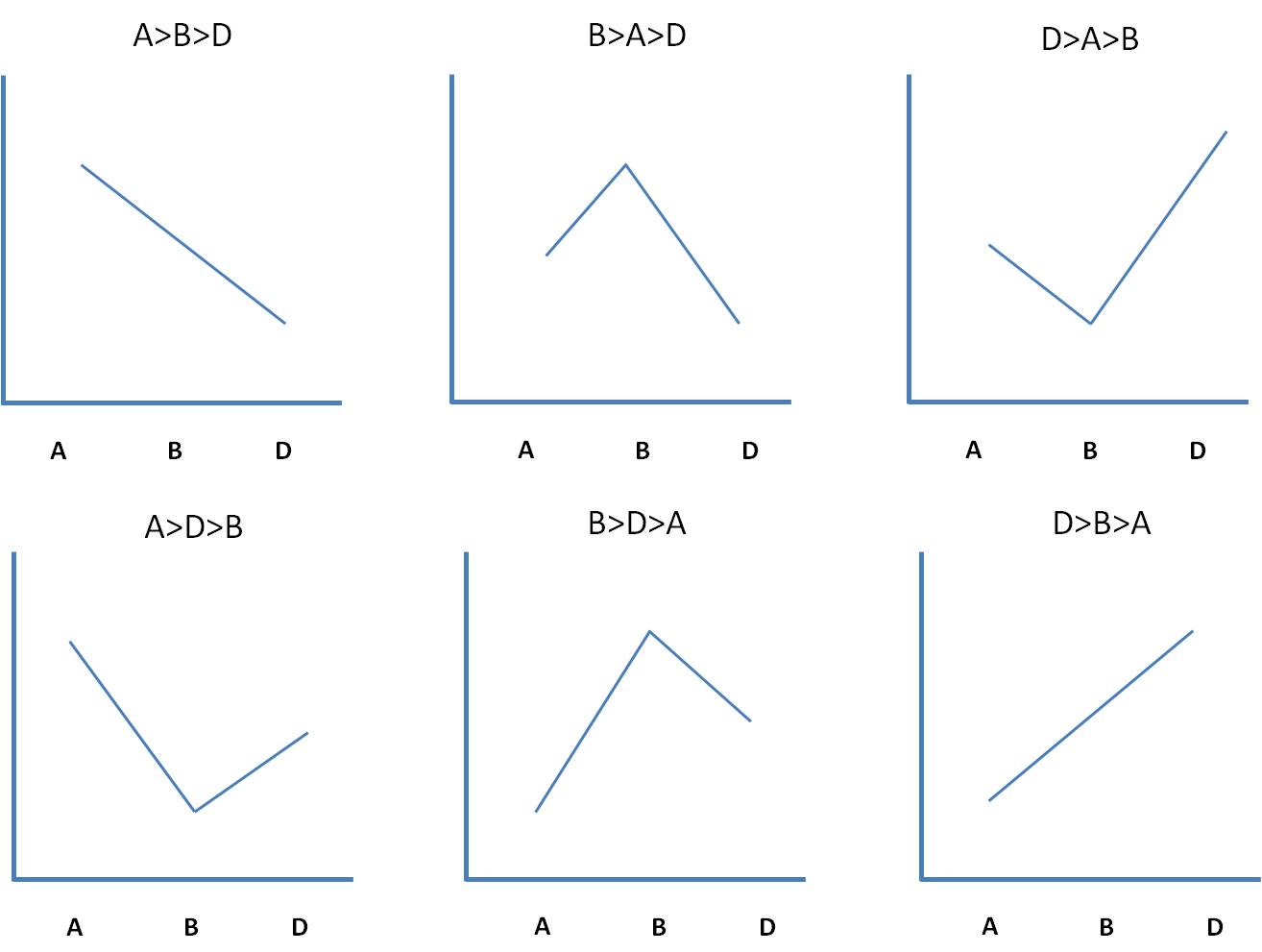


Fig 7. Examples of category three expression patterns. Six potential patterns of bias are possible expressed as A>B>D, A>D>B, B>A>D, B>D>A, D>A>B and D>B>A.

**BLAST2GO analysis to identify biotic stress related genes**

BLAST2GO analysis was performed on the global wheat coding sequence collection. We used standard parameters (outlined in Conesa et al. 2005) for this analysis running the process on a BLAST2GO server running on the CSIRO computing cluster ‘Bragg’. In total, annotations were assigned to 99,416 coding sequences. We then retrieved expression and induction bias data for sequences with biotic stress related functional descriptions to make our observations.

**References**

**Anders S, Huber W. 2010.** Differential expression analysis for sequence count data. *Genome Biol* **11**(10): R106.

**Conesa A, Götz S, García-Gómez JM, Terol J, Talón M, Robles M. 2005.** Blast2GO: a universal tool for annotation, visualization and analysis in functional genomics research. *Bioinformatics* **21**(18): 3674-3676.

**Cox MP, Peterson DA, Biggs PJ. 2010.** SolexaQA: At-a-glance quality assessment of Illumina second-generation sequencing data. *BMC Bioinformatics* **11**(1): 485.

**Desmond OJ, Edgar CI, Manners JM, Maclean DJ, Schenk PM, Kazan K. 2006.** Methyl jasmonate induced gene expression in wheat delays symptom development by the crown rot pathogen *Fusarium pseudograminearum*. *Physiological and Molecular Plant Pathology* **67**(3): 171-179.

**Langmead B, Salzberg SL. 2012.** Fast gapped-read alignment with Bowtie 2. *Nature Methods* **9**(4): 357-359.

**Li HB, Xie GQ, Ma J, Liu GR, Wen SM, Ban T, Chakraborty S, Liu CJ. 2010.** Genetic relationships between resistances to Fusarium head blight and crown rot in bread wheat (*Triticum aestivum* L.). *Theoretical and Applied Genetics* **121**(5): 941-950.

**Stephen S, Cullerne D, Spriggs A, Helliwell C, Lovell D, Taylor J. 2012.** BioKanga: a suite of high performance bioinformatics applications. *Available at ht tp://code. google. com/p/biokanga*.
