# Supplementary figures and images for: The defence‐associated transcriptome of hexaploid wheat displays homoeolog expression and induction bias
Source: Plant Biotechnol J. 2016 Nov 11;15(4):533–43. doi: 10.1111/pbi.12651 (PMC5362679; doi:10.1111/pbi.12651)

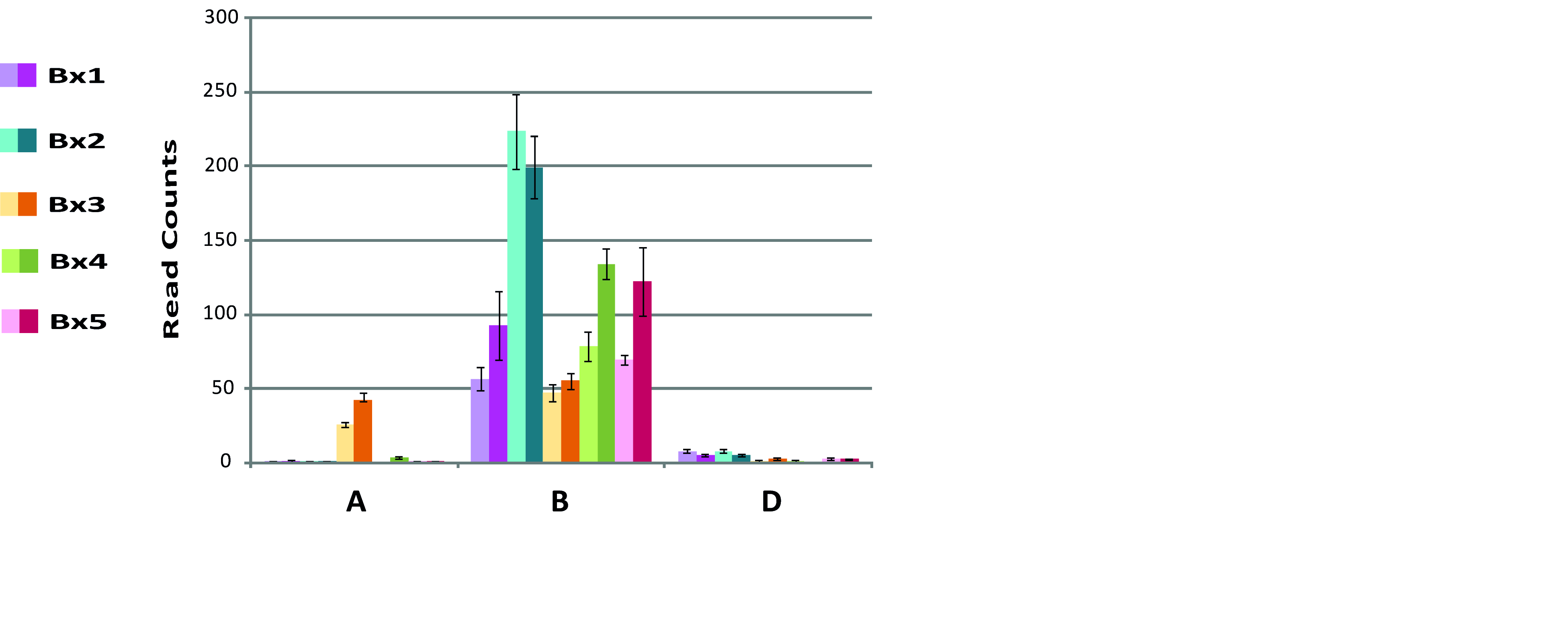

Supplement: Supplementary file 3 — Appendix S2 Testing Alignment Stringency for Differentiating Homoeologous Gene Copies. Panel A: shows expression estimates for all homoeologous copies for Bx1–5 except for the B copy of Bx3 which was found to be absent from the CSS reference. Panel B: shows expression estimates when read alignment was performed using the CSS reference with the known TaBx3B coding sequence added. [file PBI-15-533-s002.tif]

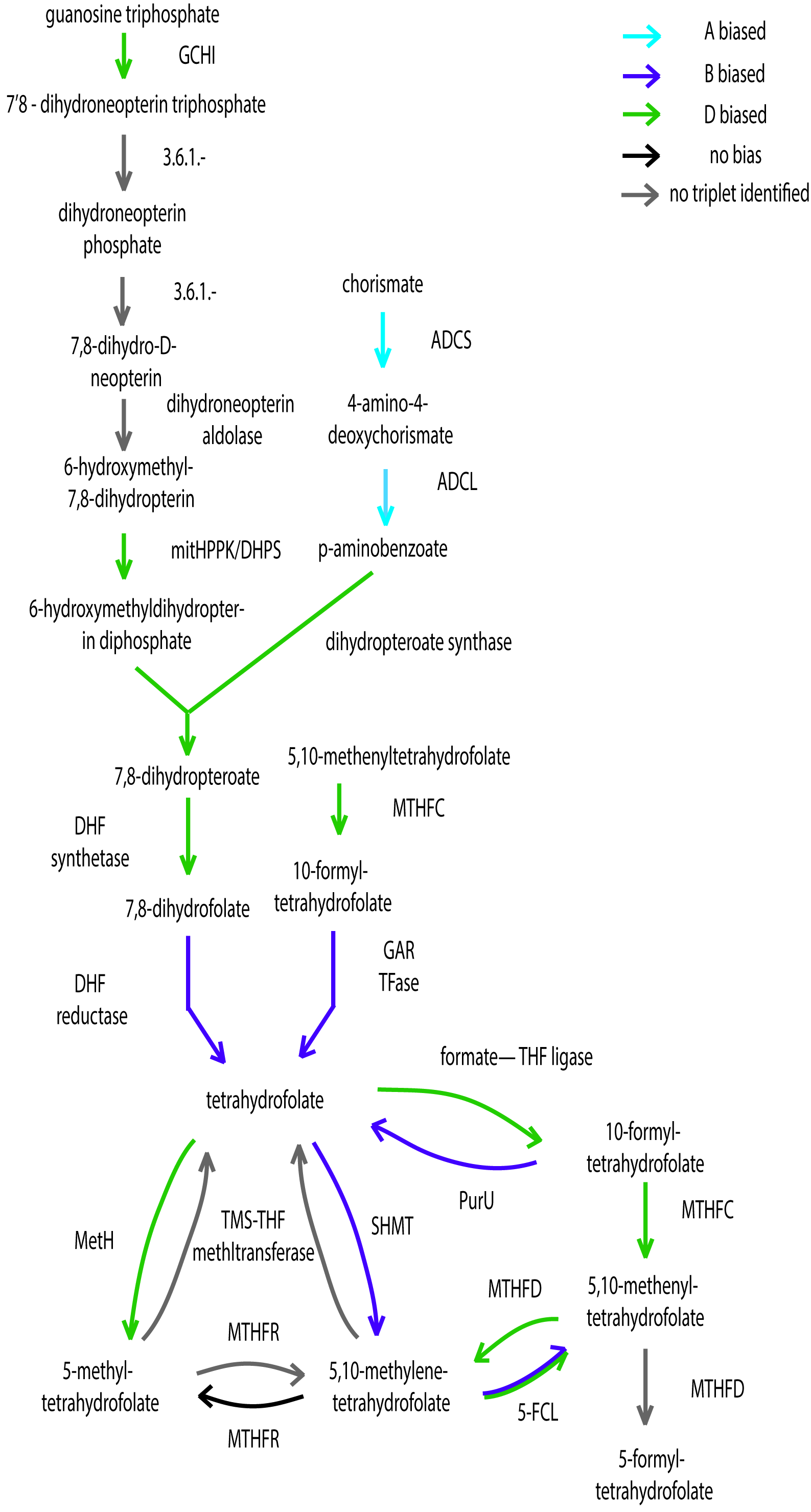

Supplement: Supplementary file 4 — Appendix S3 Homoeolog expression bias within the folate biosynthesis pathway favours B and D subgenomes. Cyan, purple and green arrows represent steps encoded by triplets displaying an expression bias towards the A, B and D subgenome homoeologs, respectively. Grey arrows represent enzymatic steps for which no triplets could be identified and black arrows represent enzymatic steps where triplets showed no bias. [file PBI-15-533-s001.tif]

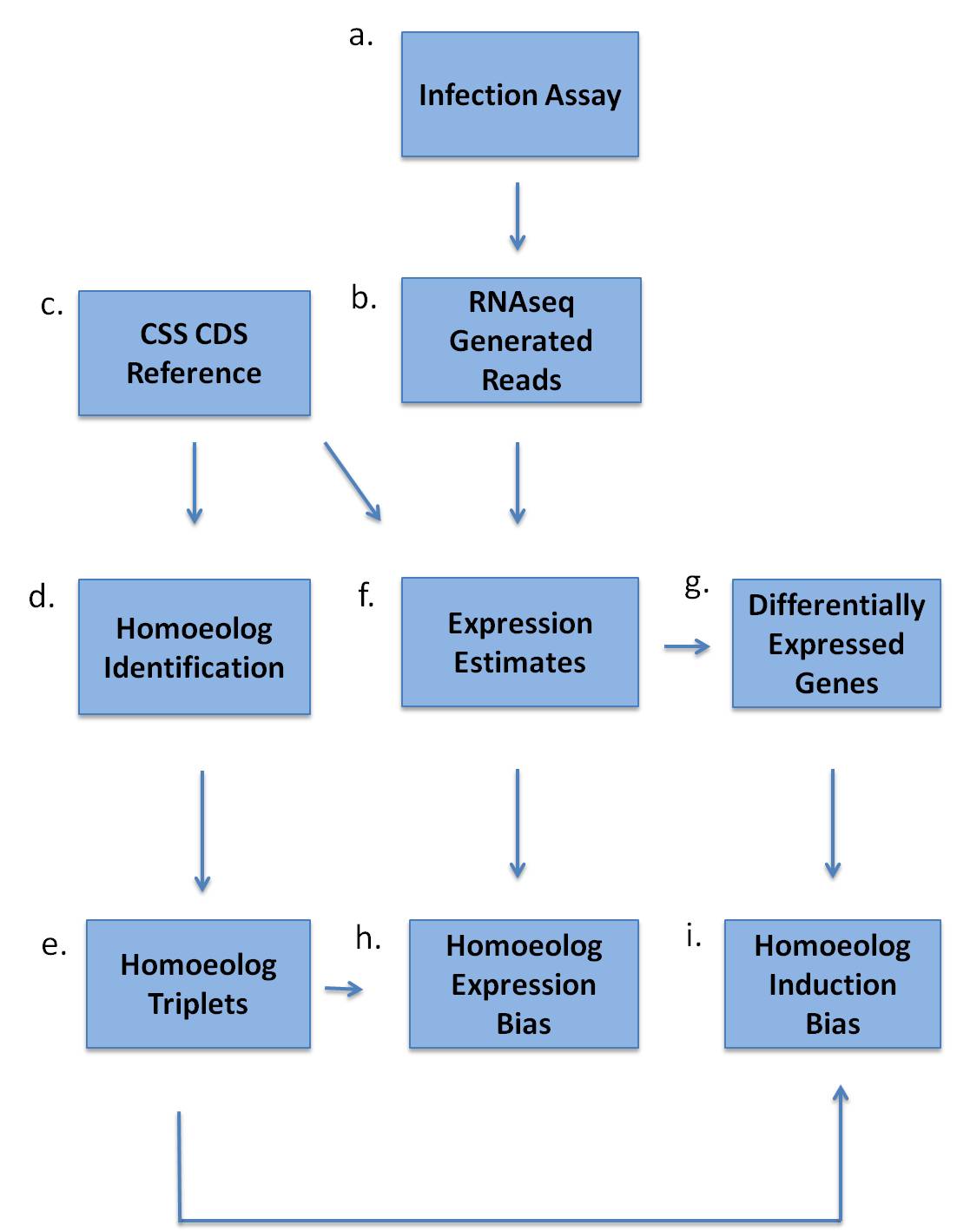

Supplement: Supplementary file 5 — Appendix S4 Graphical representation of the analysis pipeline to observe homoeolog expression bias during application of a biotic stress. Firstly, an infection assay was performed to produce mock and F. pseudograminearum inoculated wheat tissue samples (a) and RNA‐seq was performed to generate reads from transcripts (b). The wheat genome chromosomal survey sequence (coding sequence collection) (c) was utilized within a reciprocal best BLAST approach to identify homoeologs (d) with ~13 000 homoeolog triplets identified and validated (e). RNA‐seq generated reads were aligned to the wheat genome (coding sequences as reference) using Bowtie2 and counted to estimate gene expression globally (f). DESeq was used to identify genes differentially expressed between mock‐ and Fp‐treated samples (g). Homoeolog expression bias was assessed in three‐way pairwise comparison using DESeq to identify triplets in which one or more homoeologs were expressed to a significantly different level compared to the others (h). Finally, genes differentially expressed during infection which were also captured within inferred homoeolog triplets were analysed for homoeolog induction bias (i). [file PBI-15-533-s005.jpg]
